# Unveiling the molecular dynamics of a nitrile-containing 5-lipoxygenase-activating protein antagonist in primary macrophages through Raman spectroscopy

**DOI:** 10.1039/d5sc09493c

**Published:** 2026-01-09

**Authors:** Constanze Schultz, Paul Mike Jordan, Philipp Dahlke, Zehra Tuğçe Gür Maz, Erden Banoğlu, Tobias Meyer-Zedler, Michael Schmitt, Oliver Werz, Juergen Popp

**Affiliations:** a Leibniz Institute of Photonic Technology (Leibniz-IPHT), Member of Leibniz Health Technologies, Member of the Leibniz Center for Photonics in Infection Research (LPI) Albert-Einstein-Str. 9 07745 Jena Germany juergen.popp@uni-jena.de; b Institute of Pharmaceutical Chemistry, Friedrich Schiller University Jena Philosophenweg 14 07743 Jena Germany; c Department of Pharmaceutical Chemistry, Faculty of Pharmacy, Gazi University Taç Sok. No:3 Yenimahalle 06560 Ankara Turkey; d Institute of Physical Chemistry (IPC) and Abbe Center of Photonics (ACP), Member of the Leibniz Center for Photonics in Infection Research (LPI), Friedrich Schiller University Jena Helmholtzweg 4 07743 Jena Germany

## Abstract

The nitrile (–C≡N) functional group is a versatile pharmacophore motif that also serves as an intrinsic, bioorthogonal Raman tag in the silent wavenumber region (1800–2700 cm^−1^). Here, we exploit this dual functionality to track the potent nitrile-containing 5-lipoxygenase-activating protein (FLAP) antagonist BRP-685 in primary human macrophages using label-free spontaneous and stimulated Raman scattering. This approach enables direct intracellular localization at biologically relevant, low micromolar concentrations without chemical modification or external labels. Quantitative Raman imaging reveals that BRP-685 preferentially accumulates in lipid droplets, distinct from its membrane-bound target site at the nuclear envelope/endoplasmic reticulum. Multiplexed analysis with an alkyne-tagged lipid analog uncovers a unique distribution pattern, suggesting that lipid droplets act as intracellular reservoirs for highly lipophilic drugs.

## Abstract 2

The nitrile group is a versatile pharmacophore that also functions as an intrinsic, bioorthogonal Raman tag in the silent wavenumber region. This dual role allows visualization of the storage sites and sites of action of the FLAP antagonist BRP-685 within differently polarized macrophages.

## Graphical abstract

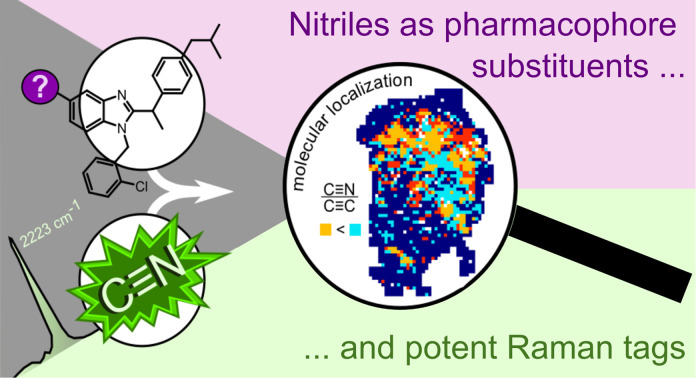

## Introduction

Despite modest abundance frequency in natural products,^1^ the nitrile functional group (–C≡N) is consistently associated with potent biological activity, often in the context of chemical defense strategies^2,3^ or signaling,^4^ and plays key roles as an intermediate in metabolic pathways^3^ in nature. Following their natural role, nitrile substituents are increasingly employed in the design of synthetic bioactive compounds as well^1^ and have also become valuable motifs in medicinal chemistry (*e.g.*, in gliptins).^5^ As a part of the pharmacophore, nitriles act as bioisosteres,^6^ notably replacing carbonyl moieties,^4^ but also serve important roles as pharmacokinetic and pharmacodynamic substituents. They typically interact with target proteins through hydrophobic interactions, covalent bonding, and, most prominently, hydrogen bonding. Water molecules participating in this hydrogen-bonding network further facilitate the association of nitrile groups with nearby amino acid residues.^5^ Such interactions are critical for the binding activity of pharmaceuticals and influence the electronic and steric properties of molecules, consequently shaping their chemical and biological behavior (*e.g.*, solubility, binding affinity, metabolic stability, bioavailability). These properties have allowed the nitrile moiety to gain momentum in drug design, with more than 70 FDA-approved drugs currently featuring nitrile groups (https://go.drugbank.com, 06/11/2025).^7^

Given their pronounced importance in pharmacology, it is particularly attractive that nitrile groups can be detected with exceptional specificity in their native environment using label-free Raman spectroscopy. Their distinct C≡N stretching vibration lies in the so-called silent wavenumber region (1800–2700 cm^−1^) of the vibrational spectrum, where virtually no other endogenous molecular vibrations occur. This spectral isolation enables unambiguous detection and quantification of nitrile moieties in complex biological matrices without the need for additional molecular tags. Owing to this property, nitrile groups are not only readily detectable when intrinsically present, but also ideally suited as minimal-size Raman tags when introduced synthetically. In contrast to bulky fluorescent labels, the compact nitrile group perturbs the molecular structure only minimally while providing spectral, biological, and chemical orthogonality – features that are highly advantageous for non-surface-enhanced Raman applications.^8^ Interestingly, the practical value of nitriles for real-world applications, particularly in drug targeting, is underappreciated, possibly due to their significantly lower intensity compared to similar alkynes.^9^ Only a few examples exist that employ molecules bearing one or more simple nitrile groups,^10–13^*i.e.*, not embedded in specially-designed extended reporter structures^16–19^ or polymer beads. Despite growing interest in bioorthogonal Raman tags, direct cellular investigation of drug distributions by non-surface-enhanced Raman remains rare,^14,15^ with neratinib being likely the only reported nitrile-containing drug.^12^ We thereby define drugs, in contrast to sensors, as molecules specifically designed for therapeutic use in humans.

In this contribution, we aim to support the molecular route of nitrile-containing drugs intracellularly in primary human macrophages, focusing on an antagonist targeting the 5-lipoxygenase-activating protein (FLAP). FLAP's subcellular loci are the nuclear envelope and associated endoplasmic reticulum (ER),^20^ where it supports the enzyme 5-lipoxygenase (5-LOX) in converting arachidonic acid (AA) into the pro-inflammatory mediator leukotriene B_4_ (LTB_4_) in innate immune cells. Through this role, FLAP contributes to the initiation and progression of inflammatory processes,^21,22^ and efforts to identify potent antagonists hold significant promise for advancing anti-inflammatory therapies.^23^

## Results and discussion

**Fig. 1:**
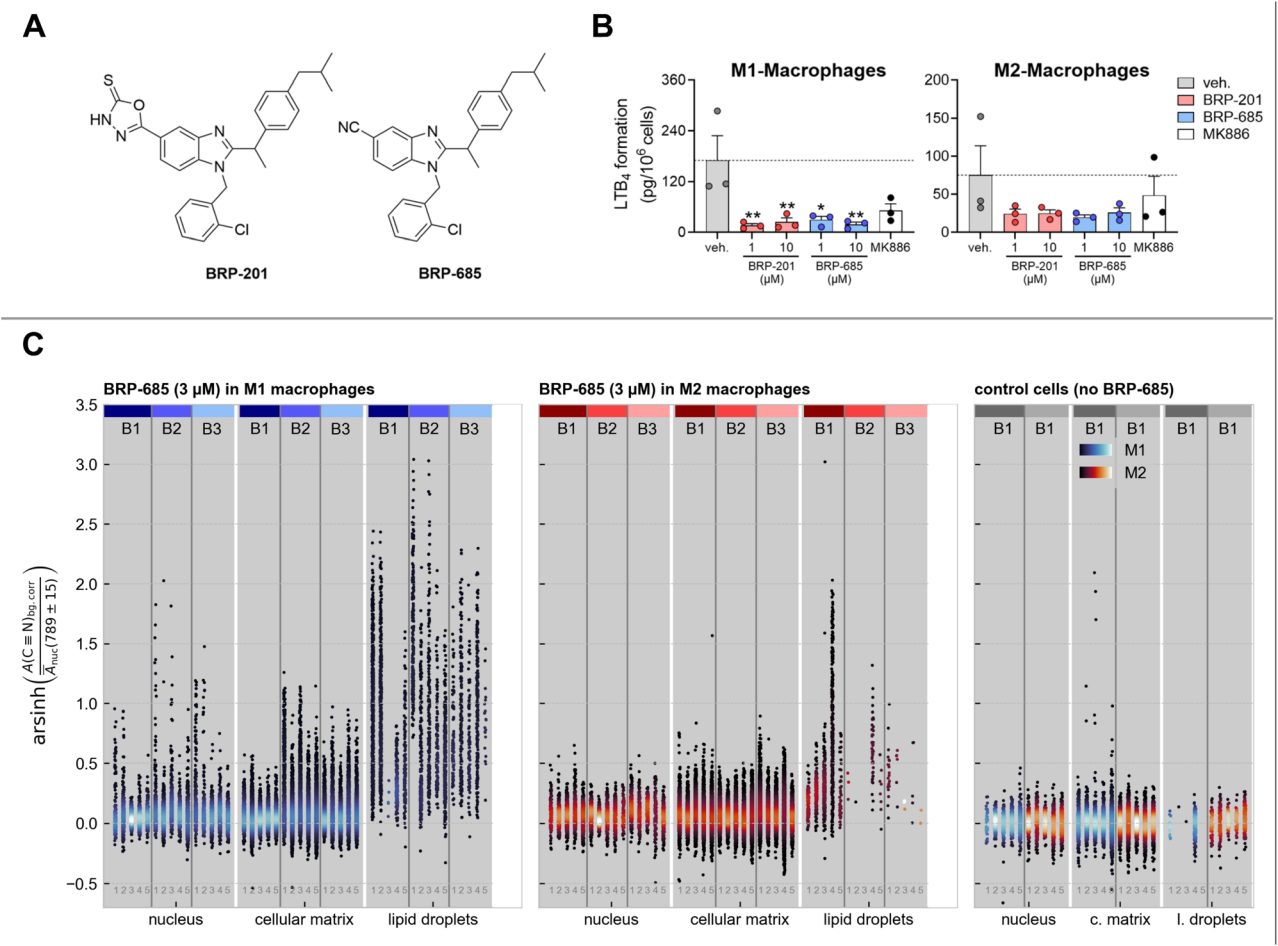
Bioactivity of BRP-201 and BRP-685 and localization of BRP-685 in human primary macrophages. (A) Structures of BRP-201 and BRP-685. (B) Human M1 or M2 macrophages (10^6^ cells per mL) were preincubated with vehicle (veh., 0.1% DMSO), 1 or 10 µM BRP-201 or BRP-685, and 0.03 µM MK886 as a positive control for 15 min before challenge with *Staphylococcus aureus*-conditioned medium (SACM, 1%) for 90 min at 37 °C. Formed LTB_4_ was quantified in the supernatants using UPLC-MS/MS. For statistical analysis, data were log-transformed and analyzed *via* one-way ANOVA with Dunnett's multiple comparison test; *p < 0.05, **p < 0.01. (C) The integrated and normalized Raman signal intensity of the nitrile band of BRP-685 (centered at (2223 ± 15) cm^−1^) is shown for different cellular compartments. All measurements of (C) were performed using spontaneous Raman microspectroscopy. Results for BRP-685-treated M1 macrophages (blue) are displayed in the left panel of subfigure C, while M2 macrophages (orange) are shown in the middle panel of subfigure C. Each cell type includes three independent batches (B1, B2, B3), with 5 individual cells per batch (labels 1–5). Batches are indicated by vertical gray lines and colored patches at the top; identical colors represent the same batch, while different colors indicate separate batches. Data points sharing the same *x*-position within a batch correspond to the same cell, whereas each scatter point represents a distinct pixel within the same measured cell, capturing spatial heterogeneity across the defined cellular region. The highest BRP-685 signal is detected in lipid droplets, particularly abundant in M1 macrophages. Nucleus and general cellular matrix regions show comparable mean signal levels, with slightly higher signal spread observed in the cellular matrix for both macrophage types. The point density in the *y*-direction is encoded within the given colormap and normalized across the figure. The right panel shows control data from untreated cells, with M1 and M2 macrophages color-coded as in the treated samples. For more details, see the text or Method section in the SI.

Recently, we developed the benzimidazole-based BRP-201 and its nitrile-containing derivative BRP-685 as potent FLAP antagonists (Fig. 1A).^24,25^ The synthetic route to BRP-685 is shown in Scheme S1. Intriguingly, we found that BRP-201 acts as a lipid mediator class-switching agent by reducing pro-inflammatory LTB_4_ production but elevating the formation of pro-resolving lipid mediators (SPM) in human macrophages.^23^

Despite this favorable impact on SPM formation, the underlying molecular basis of this action is obscure.

### Subcellular mapping of BRP-685 *via* spontaneous Raman micro-spectroscopy

In this contribution, we harness the unique potential of Raman spectroscopy for label-free, chemically specific, and spatially resolved tracking to elucidate the intracellular fate and molecular dynamics of the nitrile-containing FLAP antagonist BRP-685 in primary human macrophages.

Consistent with previous studies,^21^ both BRP-685 and BRP-201 (Fig. 1A) suppress LTB_4_ formation in exotoxin-stimulated human primary M1/M2 macrophages, acting more effectively in M1 cells with higher FLAP expression (Fig. 1B). Raman imaging followed by supervised segmentation was used to examine BRP-685 distribution and accumulation across cellular areas. Based on the spatial subcellular distribution of various Raman modes, a distinction of the nucleus, lipid droplets, and other cell compartments (in the following called “cellular matrix”) was deemed feasible. Therefore, we manually annotated 7 images (Fig. S1A) and trained a Random Forest Classifier (for more details, see Methods section). The segmentation efficiency was then verified for the cells treated with 3 µM BRP-685 by the analysis of characteristic Raman bands and their occurrence within the segmented classes (Fig. S2). The relative abundance of the nitrile-tag, derived from integration of the 2223 cm^−1^ band area (nitrile-stretching vibration), was used to extract the accumulation position of BRP-685 across cellular compartments (Fig. 1C). To mitigate the effect of the underlying combinatorial water band^26,27^ in the silent wavenumber region, only the area under the spectrum and a linear function between both bounds of integration was considered for the silent wavenumber band. To ensure comparability between the results of different regions and individual cells, the integration results were normalized by the mean intensity of the band at (789 ± 15) cm^−1^ (cytosine and O–P–O, DNA^28–30^) in each cell's nuclear region.^31^ Accordingly, the ratio *A*(C≡N)_bg.corr_·[*Ā*_nuc_ ((789 ± 15) cm^−1^)]^−1^ represents the BRP-685 content per cell and can even assume negative values in cases where only noise is present in the integrated silent wavenumber region. Given the wide dynamic range of values across subcellular compartments, the *y*-axis spread was compressed using an inverse hyperbolic sine (arsinh) transformation (Fig. 1C), which, unlike the conventional logarithm, remains defined for negative band ratios.

The intensity of the nitrile's Raman band is significantly elevated within the lipid droplets, whereas the mean intensity of the nitrile's Raman band in the nucleus and cellular matrix does not differ for BRP-685-treated M1/M2 cell types (Fig. S3). It appears that the BRP-685-containing lipid droplets accumulate in proximity to the perinuclear region and the associated ER, where FLAP resides. The higher spread of the data within the cellular matrix area compared to the nucleus indicates a non-homogeneous distribution of BRP-685 across the cell (Fig. S4). Both key features, the accumulation of BRP-685 in lipid droplets and the “broader” distribution of values along the *y*-axis for the cellular matrix as opposed to the nucleus in Fig. 1C, indicating BRP-685 incorporation into non-nuclear regions, were confirmed by comparison with untreated control cells, which showed neither characteristic (Fig. 1C). The internalization of BRP-685 into the cell body, rather than its nonspecific attachment to the cell surface, was confirmed by *z*-projections, retrieved either from small area images of lipid droplets captured by spontaneous Raman microscopy (Fig. S5) or as whole-cell *z*-scans acquired by fast stimulated Raman scattering (SRS) (Fig. S6).

### Evaluation of BRP-685's vibrational signatures for label-free Raman imaging applications in complex environments

**Fig. 2:**
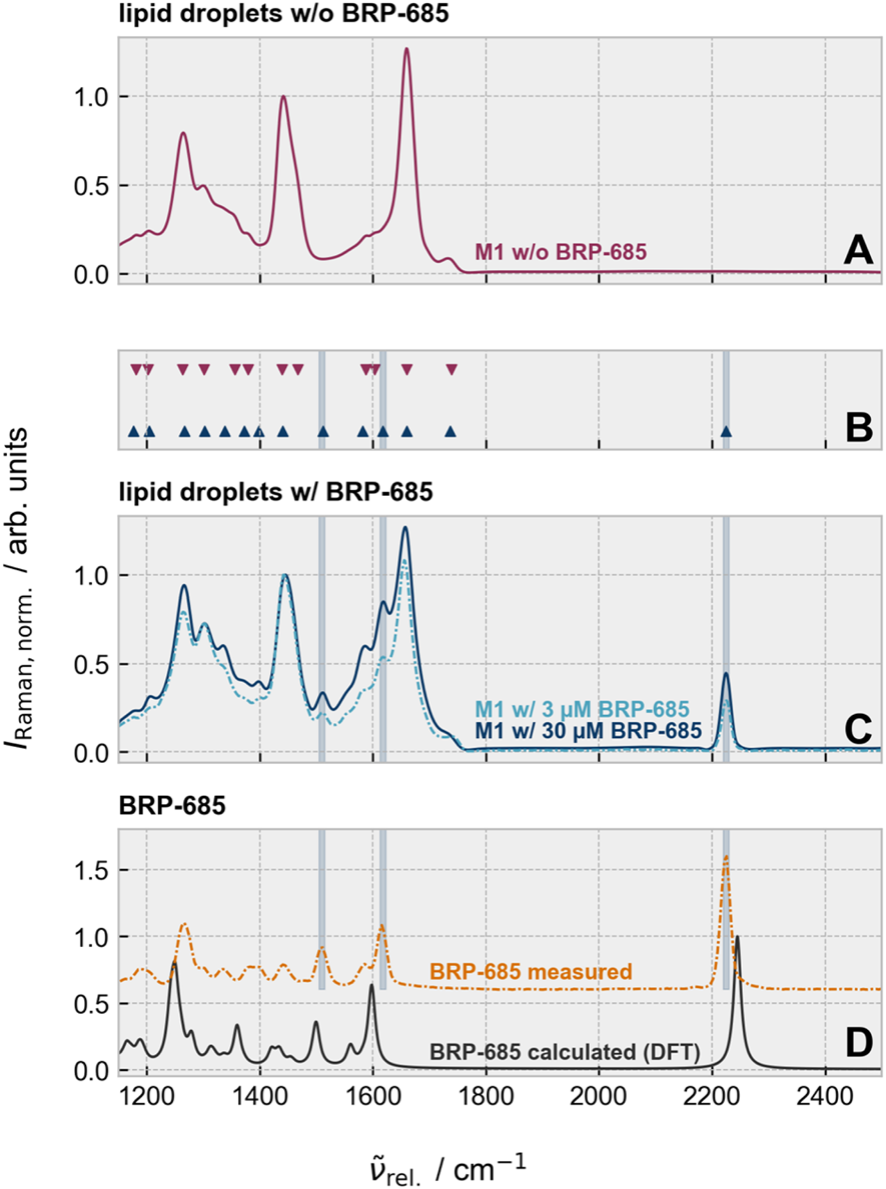
Comparison of the mean Raman spectrum within the lipid droplet region of a single cell for M1 macrophages incubated without BRP-685 (A) and with 30 µM or 3 µM BRP-685 (C). The band shape between (A and C) varies. The positions where new bands appear were identified by a peak finding algorithm (B), and the positions of the most severe variation were highlighted by colored bars. The newly appearing Raman bands on cells that were treated with BRP-685 can be assigned to the drug itself, as a comparison with a theoretically calculated spectrum (DFT) or a spectrum from a particle in a cell culture shows (D). For all shown Raman spectra, the maximum intensity in the displayed wavenumber range was scaled to 1. For more information on the DFT calculation and peak finding algorithm, please refer to the text and SI. All measured spectra shown in figure were recorded by spontaneous Raman scattering.

Given the highest intensities (local concentrations) of the nitrile's stretching band within the lipid droplets, we determined which other spectral features of the drug could be identified by Raman microscopy. Fig. 2 compares the Raman spectral features within the fingerprint wavenumber and silent wavenumber region of lipid droplets in M1 cells treated without (Fig. 2A) and with (Fig. 2C) BRP-685. Besides, the silent wavenumber band at 2223 cm^−1^, the peak picking algorithm explicitly detected two other Raman bands at 1618 cm^−1^ and 1510 cm^−1^ that were absent in the untreated but present in M1 macrophages treated with 3 µM or with 30 µM BRP-685 (Fig. 2B). Because BRP-685 is sufficiently small, its vibrational modes can be assigned using quantum chemical calculations.^32^ Density functional theory (DFT) calculations attributed the observed bands to ring vibrations of the aromatic core, specifically to those of the benzimidazole (1510 cm^−1^) and phenyl (1618 cm^−1^) moieties (Fig. S7), which are also integral to the structure of BRP-201. A vibrational mode at 1255 cm^−1^, associated with the benzimidazole unit, was present in both the DFT-calculated and particle-extracted spectra of BRP-685 (Fig. 2D), although it could not be reliably distinguished from the biological background in the cellular context. Although the shared structural features of BRP-201 and BRP-685 principally allow their detection in the vibrational fingerprint region, the inherently low intensity of most fingerprint Raman modes results in pronounced signal attenuation at physiologically relevant concentrations (3 µM) and limits conventional label-free Raman detection in biomedical and pharmaceutical research. BRP-685's intrinsic nitrile group, however, produces a sharp, intense, and bioorthogonal Raman signal in the silent wavenumber region, allowing reliable detection even at low concentrations in the cellular matrix, which effectively overcomes the sensitivity limitations of fingerprint-based detection. Consequently, BRP-685 is not only a potent FLAP inhibitor but also an exceptionally suitable molecular probe for Raman-based biomedical imaging, as further demonstrated for large-area screening *via* SRS in the SI (Fig. S8).

### Multiplex analysis of BRP-685 and an alkyne-tagged lipid for refined subcellular detection

Building on the observed enrichment of BRP-685 in lipid droplets (Fig. 1), we sought to directly compare its subcellular distribution with that of a classical lipid analog. Since spontaneous Raman scattering generally provides insufficient speed for imaging weak vibrational modes in live-cell samples, we employed stimulated Raman scattering (SRS) microscopy,^33,34^ which offers higher signal levels and fast imaging capabilities. To this end, we performed multiplexed imaging experiments using alkyne-functionalized 17-octadecynoic acid (17-ODYA) alongside the FLAP inhibitor BRP-685. For optimal signal detection, BRP-685 was applied at a concentration of 30 µM, while 17-ODYA was used at 417 µM,^35–37^ delivered *via* fatty acid-free bovine serum albumin. Cells were treated under three conditions: (i) 17-ODYA alone, (ii) BRP-685 alone, and (iii) combined treatment. Detailed protocols are provided in the SI.

**Fig. 3:**
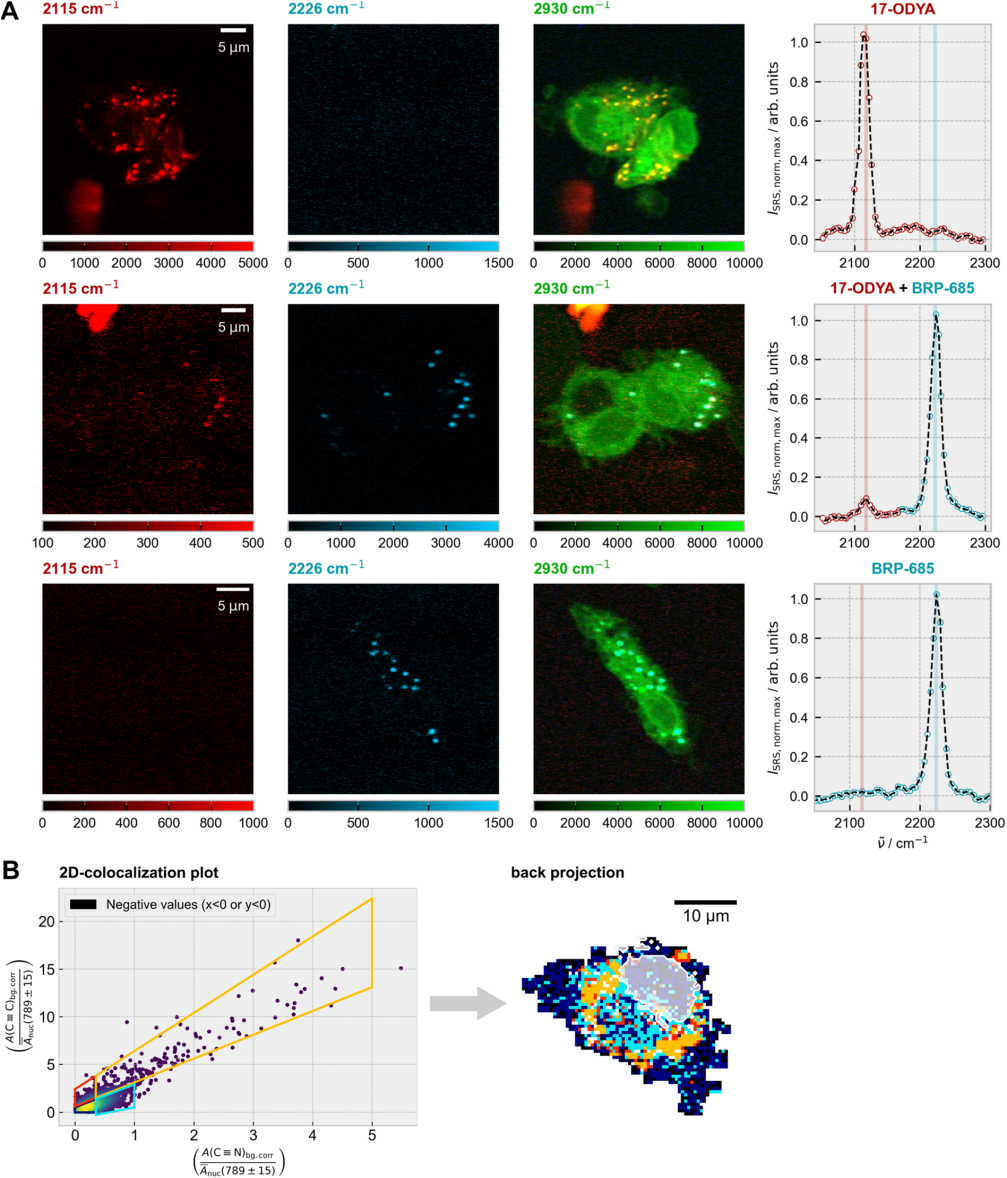
Subcellular colocalization analysis of 17-octadecynoic acid (17-ODYA) and BRP-685. (A) Representative SRS images of M1 macrophages showing the C≡C stretching mode of 17-ODYA (red, left), the C≡N stretching mode of BRP-685 (blue, middle), and the CH_3_ stretching mode (green, right). To eliminate non-specific background, the off-resonant signal at 2500 cm^−1^ was subtracted from each of the displayed images. Three treatment conditions are displayed: 17-ODYA only (top), 17-ODYA + BRP-685 (middle), and BRP-685 only (bottom). The far-right column shows representative SRS spectra of the silent wavenumber region from lipid droplet areas. (B) Subcellular colocalization analysis of the C≡C and C≡N bands based on spontaneous Raman spectra, as described in the Methods section (SI). The area under the respective Raman bands was normalized by the DNA signal in the nucleus. In the back projection image, pixels were colored according to the corresponding segments in the scatter plot. Pixels with negative values in either the *x*- or *y*-coordinate of the scatter plot are shown in black. The nucleus is indicated by a semi-transparent white contour overlaid on the back-projection image.

Both tagged compounds were unambiguously resolved within the M1 cells' silent wavenumber Raman region (Fig. 3A) *via* their triple bond stretching vibrations at 2115 cm^−1^ (C≡C for 17-ODYA) and 2223/2226 cm^−1^ (C≡N for BRP-685). Overlay of the target signals with the CH_3_-stretching signal (2930 cm^−1^) confirmed the presence of both substances inside the cells.

**Fig. 4:**
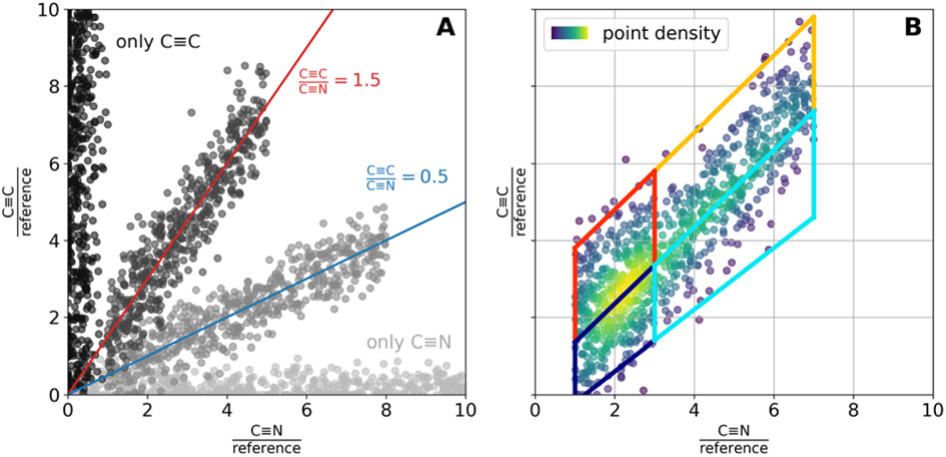
Modelling description for the performed colocalization analysis. (A) Plotting the band intensities for two bands of interest for each measured spot in a scatter plot allows an identification of pixels with similar composition with respect to the investigated bands. For more details please see the text. (B) For the generation of Fig. 3 and S9, the scatter cloud was divided into four regions, indicated by the trapezoidal shapes in (B). Pixels assigned to each region were colored according to the corresponding patch when back projected onto the original pixel grid, enabling visualization of the spatial distribution of chemically similar pixels.

A more elaborate evaluation was performed by spontaneous Raman scattering on a single cell level using a 2D colocalization analysis as described in the Methods section (Fig. 3B) The modeling description is given by Fig. 4. The colocalization plot (Fig. 3B, left) shows a high degree of colocalization between both compounds, with no on-axis signal detectable within the cell area, which would indicate single-substance presence. An evaluation in the silent wavenumber region thereby further minimizes spectral misinterpretation from overlapping cellular background signals.^38,39^ To further dissect the degree and nature of colocalization, the scatter plot was partitioned into four segments, each representing a specific relationship between the C≡C and C≡N signal intensities. Interestingly, the back-projection onto the original pixel grid (Fig. 3B, right) showed no random distribution, but a clear confinement to spatial areas. Given the ratio of C≡C to C≡N signals in the lipid droplet areas (orange, Fig. 3B), regions adjacent to the lipid droplets displayed a higher ratio of C≡N to C≡C (cyan), indicating relative enrichment of BRP-685. Both regions were distinct from areas with low signal intensities from either component (red or dark blue). Further supportive examples are provided in the SI (Fig. S9).

## Conclusions

Conclusively, we demonstrated that the preferential intracellular target compartment site of the FLAP antagonist BRP-685 (and BRP-201) in macrophages is lipid droplets, which contrasts with its site of action, namely the nuclear envelope/ER, where FLAP resides. This indicates that upon cellular uptake of BRP-685, the compound does not directly access the target protein but instead, as a highly lipophilic drug, first accumulates in lipid droplets. This gives rise to the idea that upon drug application, BRP-685 might then be transferred in a delayed manner to the perinuclear region and the ER (Fig. 3B), where membrane-bound FLAP facilitates the delivery of AA to 5-LOX as substrate for LTB_4_ formation. In such a scenario, lipid droplets may act as intracellular delivery devices for highly lipophilic drugs to accomplish the effective supply of poorly soluble drugs within the cell to (membrane organelle-bound) target proteins. These important insights were made possible by Raman spectroscopy, which enabled chemically specific and spatially resolved tracking of BRP-685 in its native intracellular environment. In a broader context, this work has highlighted the versatility of the nitrile functional group to operate both as a pharmacophore substituent and a kind of “label-free”-tag for distinguishing specific molecular targets from a diverse biomolecular background matrix. The findings of this study hold significant promise, not only for drug-localization studies and strategies to elucidate intracellular distribution, but also for establishing the largely untapped nitrile functional group – as opposed to the widely used alkyne tag – as a powerful Raman reporter. Moreover, the possibility of label-free vibrational identification enables unbiased detection strategies that can compete with gold-standard fluorescence imaging with external labels, while also reinforcing the choice of nitrile groups as substituents in the design of novel drug candidates.

## Experimental

### Isolation of monocytes and differentiation and polarization to macrophages

Monocytes were isolated from leukocyte concentrates obtained from freshly withdrawn peripheral blood of healthy adult male and female donors, provided by the Institute of Transfusion Medicine, University Hospital Jena, Germany. The experimental protocol was approved by the ethical committee of the University Hospital Jena (approval no. 5050-01/17). Informed consent was obtained from all human subjects. All methods were performed in accordance with the relevant guidelines and regulations. This protocol is described in detail in Günther *et al.*^40^ In brief, leukocyte concentrates were mixed with dextran (derived from *Leuconostoc* spp., *M*_W_ ∼40 000, Sigma-Aldrich) for sedimentation of erythrocytes. The supernatant was centrifuged on lymphocyte separation medium (Histopaque®-1077, Sigma-Aldrich). The peripheral blood mononuclear cell (PBMC) fraction was seeded in RPMI 1640 (Sigma-Aldrich) containing 10% (v/v) heat-inactivated fetal calf serum (FCS), 100 U per mL penicillin, and 100 µg per mL streptomycin in cell culture flasks (Greiner Bio-one, Frickenhausen, Germany) and incubated for 1.5 h at 37 °C and 5% CO_2_ for adherence of monocytes. For differentiation of monocytes to macrophages and subsequent polarization towards M1- and M2-monocyte-derived macrophages (MDM), we used published criteria and procedures.^40,41^ Thus, M1-MDM were generated by incubating monocytes with 20 ng per mL GM-CSF (Peprotech, Hamburg, Germany) for 6 days in RPMI 1640 supplemented with 10% FCS, 2 mmol per L l-glutamine (Biochrom/Merck, Berlin, Germany), and penicillin–streptomycin (Biochrom/Merck), followed by treatment with 100 ng per mL lipopolysaccharide and 20 ng per mL interferon-γ (Peprotech) for another 24 h. M2-MDM were obtained by incubating monocytes with 20 ng per mL M-CSF (Peprotech) for 6 days and subsequent polarization by treatment with 20 ng per mL interleukin (IL)-4 (Peprotech) for 48 h.

### Incubation of macrophages

For lipid mediator production, polarized M1- and M2-MDM (1 × 10^6^ mL^−1^) were seeded in 12-well plates in PBS buffer plus 1 mM CaCl_2_. Cells were then treated with 1 and 10 µM BRP-201, BRP-685 or vehicle (0.1% DMSO), as well as with the FLAP antagonist MK886 (0.03 µM, reference compound, Cayman Chemical/Biomol GmbH, Hamburg, Germany) for 15 min and then incubated with 1% *Staphylococcus aureus*-conditioned medium (SACM) for 90 min at 37 °C; SACM was produced as previously described.^42^ The supernatants (1 mL) of these incubations were transferred to 2 mL of ice-cold methanol containing deuterium-labeled internal standards (200 nM d_8_-5S-HETE, d_4_-LTB_4_, d_5_-LXA_4_, d_5_-RvD2, d_4_-PGE_2,_ and 10 µM d_8_-AA; Cayman Chemical/Biomol GmbH, Hamburg, Germany) to facilitate quantification and sample recovery. For Raman spectroscopic experiments, polarized M1- and M2-MDM (1 × 10^6^ mL^−1^) were seeded on cover slides in PBS buffer plus 1 mM CaCl_2_. Then, cells were stimulated with different concentrations of BRP-685 or vehicle (0.1% DMSO). For the 17-octadecynoic acid (17-ODYA) experiments, cells were seeded on cover slides in RPMI 1640 supplemented with 10% FCS, 2 mmol per L l-glutamine, 100 U per mL penicillin, and 100 µg per mL streptomycin and incubated with 417 µM 17-ODYA for 30 min at 37 °C. The 17-ODYA solution used for incubation was prepared as recently reported.^35–37^ Afterwards, cells were treated with indicated concentrations of BRP-685 or vehicle (0.1% DMSO) for 30 min. Cell experiments for Raman spectroscopic investigations were stopped on ice after the indicated times, and then the cell supernatant was discarded. Afterwards, cells were fixed with 4% paraformaldehyde (PFA) for 20 min at room temperature and then washed three times with PBS.

### Lipid mediator analysis by UPLC-MS/MS

Solid phase extraction (SPE) and sample preparation for UPLC-MS/MS analysis were conducted by adapting published criteria.^43^ Briefly, samples were kept at −20 °C for 60 min to allow protein precipitation. After centrifugation (1200 g, 4 °C, 10 min), 8 mL acidified H_2_O was added to the supernatants (final pH = 3.5), and samples were subjected to SPE. Solid phase cartridges (Sep-Pak® Vac 6cc 500 mg/6 mL C18; Waters, Milford, MA, USA) were equilibrated with 6 mL methanol and 2 mL H_2_O. Then, samples were loaded onto columns and washed with 6 mL H_2_O and additionally 6 mL *n*-hexane. Lipid mediators (LM) were eluted with 6 mL methyl formate, the eluates were brought to dryness using an evaporation system (TurboVap LV, Biotage, Uppsala, Sweden), and lipid mediators (LM) were resuspended in 100 µL methanol/water (50 : 50, v/v) for UPLC-MS/MS analysis. The LM profile was analyzed with an Acquity™ UPLC system (Waters, Milford, MA, USA) and a QTRAP 5500 Mass Spectrometer (ABSciex, Darmstadt, Germany) equipped with a Turbo V™ Source and electrospray ionization. LM were separated using an ACQUITY UPLC® BEH C18 column (1.7 µm, 2.1 × 100 mm; Waters, Eschborn, Germany) at 50 °C with a flow rate of 0.3 mL min^−1^ and a mobile phase consisting of methanol/water/acetic acid of 42/58/0.01 (v/v/v) that was ramped to 86/14/0.01 (v/v/v) over 12.5 min and then to 98/2/0.01 (v/v/v) for 3 min.^44^ The QTrap 5500 was operated in negative ionization mode using scheduled multiple reaction monitoring coupled with information-dependent acquisition. The scheduled multiple reaction monitoring window was 60 s, optimized LM parameters were adopted,^44^ and the curtain gas pressure was set to 35 psi. The retention time and at least six diagnostic ions for each LM were confirmed by means of an external standard (Cayman Chemical/Biomol GmbH, Hamburg, Germany). Quantification was achieved by calibration curves for each LM. Linear calibration curves were obtained for each LM and gave *r*^2^ values of 0.998 or higher (for PUFA 0.95 or higher). Additionally, the limit of detection for each targeted LM was determined.^44^ The identity of low-abundance analytes was confirmed by fragmentation pattern matching by re-analysis using a QTrap 7500 mass spectrometer (Sciex, Framingham, MA, USA) controlled by SCIEX-OS, and comparing the enhanced product ion scans of the biological sample with those of authentic standards.

### Vibrational Raman spectroscopy

#### Spontaneous Raman microspectroscopy

As previously described,^31^ hyperspectral Raman maps of cells were acquired with an upright confocal Raman microscope (alpha300, WITec Wissenschaftliche Instrumente und Technologie GmbH, Ulm, Germany). Cells were prepared as described above and covered with phosphate-buffered saline (PBS) for measurement. The sample was illumined with a green laser (514 nm, Fandango, Cobold AB, Stockholm, Sweden) that was focused onto the sample using a 60×/1.0 NA water dipping objective (CFI Apo NIR, Nikon, Tokyo, Japan). The excitation power was set to 25 mW in the beam path, which resulted in a power of 22 mW at the objective's front lens. The scattered signal was collected by a 100 µm-multimode optical fiber, guided to the spectrometer (UHTS300, WITec Wissenschaftliche Instrumente und Technologie GmbH, Ulm, Germany) diffracted by a blazed optical grating (600 g mm^−1^, (BLZ = 500 nm, spectral center: 576.964 nm)) and detected by a cooled back-illuminated CCD camera (DV401A, Oxford Instruments Andor Ltd, Belfast, UK). The achievable spectral resolution for this configuration is 0.135 nm, which corresponds to about 3 cm^−1^ to 5 cm^−1^.

Hyperspectral maps of cells were measured with a spatial resolution of 0.5 µm per px and a pixel dwell time of 0.5–1.0 s. For a detailed overview of the measured samples by Raman microspectroscopy, please see Table S1 in the SI.

#### Stimulated Raman scattering (SRS) imaging

SRS images were recorded with a commercial multiphoton microscope optimized for nonlinear coherent Raman scattering (Leica Stellaris 8 CRS, Leica Microsystems GmbH, Wetzlar, Germany) as earlier described.^31,45^ A shot-noise-limited laser system (picoEmerald, APE Angewandte Physik und Elektronik GmbH, Berlin, Germany) was employed for SRS measurements within the CRS setup. The laser provided two laser beams with synchronized spatial and temporal overlap: a Stokes beam (1031.1 nm, 2 ps pulse duration, 80 MHz, 300 mW) from the main oscillator and a tunable pump beam (700–990 nm, 80 MHz, 150 mW) from the integrated optical parametric oscillator (OPO). Given this configuration, a selective excitation of individual Raman vibrational modes across a spectral range of 400 cm^−1^ to 4500 cm^−1^, with a resolution of approximately 10 cm^−1^, became possible. For SRS image acquisition, the Stokes beam was additionally modulated by the laser system's in-built electro-optic modulator (modulation frequency: 20 Hz). A water-immersion IR objective (HC PL IRAPO 40×/1.10 WATER, Leica Microsystems GmbH, Wetzlar, Germany) was used to focus the aligned beams onto the sample, and signal detection in the transmission path was carried out using a 1.4 NA oil immersion condenser placed in the buffer. The SRS signal was detected as stimulated Raman loss (SRL) in forward direction by lock-in amplification.

Single plane images were measured with a speed of 200 Hz (7.7 µs per px when using 512 px × 512 px), a frame averaging of 8, and 30% laser attenuation as used in Fig. 3 and S8. The *z*-scan in Fig. S6 was recorded with a speed of 400 Hz (6.3 µs per px, using 256 px × 256 px × 111 px), a frame averaging of 4, and 30% laser attenuation. Images for spectral retrieval (*λ*-scan of the pump wavelength) in Fig. 3 were recorded with 200 Hz (7.7 µs per px when using 512 px × 512 px), a frame averaging value of 8, and a laser attenuation of 30% (BRP-685 + 17-ODYA), 40% (BRP-685) or 50% (17-ODYA). To mitigate the effect of laser power variations chosen according to the sample condition, the retrieved spectra were normalized before plotting.

### Quantum chemical calculation of BRP-685

The theoretical calculation of the vibrational Raman spectrum of BRP-685 was accomplished in ORCA 6.0.0 (ref. 46 and 47) using a double polarized Karlsruhe valence triple-zeta basis set (def2-TZVPP)^48^ along with the B3LYP-hybrid functional^49,50^ and Grimme's DFT-D3(BJ) Becke-Johnson dispersion correction.^51,52^ A RIJCOSX^53^ approximation with Weigend's def2/J auxiliary basis set^54^ for coulomb and exchange integrals was used in addition to help streamline the calculation procedure. Polarizability and frequency calculations were performed at the optimized geometry, confirming the final structure by the absence of imaginary frequencies.

Raman intensities (*I*_*j*_) were eventually retrieved from the provided Raman activities (*S*_*j*_) utilizing eqn (1)^55^ and a temperature of *T* = 298.15 K (*ϑ* = 25 °C). While *I*_*j*_ denotes the Raman intensity of the *j*^th^ band, *ν̃*_0_ was set to the wavenumber of the incident laser beam (*λ* = 514 nm, *ν̃*_0_ = 19 455.3 cm^−1^), which we also used for the cell experiments. *ν̃*_*j*_ is the wavenumber of *j*^th^ Raman band, and *h* (Planck constant), *c* (speed of light), and *k*_B_ (Boltzmann constant) are natural constants. To minimize deviations between the calculated and measured spectra, an estimated scale factor for the frequency axis of 0.9626 was used.^56^ To obtain a spectrum that is comparable to measured spectra, the bands were broadened with a Lorentzian profile assuming a hypothetical FWHM of 15 cm^−1^.1
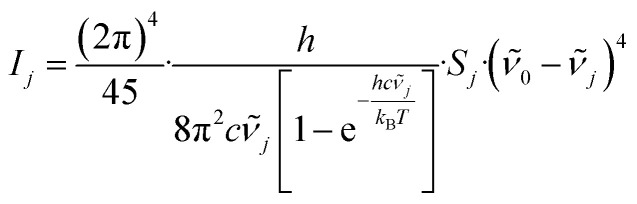


### Data processing

#### Pre-processing

Spontaneous Raman spectra hypercubes were imported into 
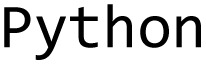
 (version: 3.10.9). First, a spike correction employing the Whittaker and Hayes approach^57^ with a threshold factor of 37 was conducted to remove sharp artifacts. Afterward, the spectra were subjected to a background correction using a sectioned SNIP correction^58,59^ (3150–2600 cm^−1^: 100 iterations, 2800–1800 cm^−1^: 7 iterations, 1900–550 cm^−1^: 100 iterations). The corrected regions were then merged to retrieve the full spectrum. Any mutual imprecisions to the wavenumber axis were eventually adjusted based on shifts calculated between measured and known wavenumber positions of the bands of 4-acetaminophen.^65^ The data were subsequently resampled onto a new, evenly spaced set of wavenumbers. In this case, the new wavenumber axis spans from 600 cm^−1^ to 3050 cm^−1^ with increments of 2 cm^−1^ using 
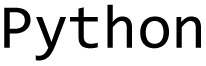
's 
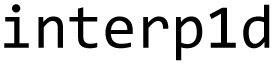
 function (from 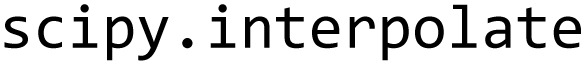).

#### Calculation of peak areas

Raman band integration images and the determination of the relative abundance were determined from the spontaneous Raman data by calculating the area under the spectrum in a given wavenumber range. Therefore, a trapezoidal numerical integration method (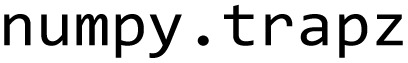), integrating the intensity values with respect to the corresponding wavenumber axis, was applied in 
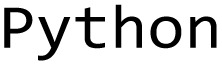
.

#### Segmentation of cell areas

The segmentation of cellular regions (nucleus, lipid droplets, other cellular areas, and the surrounding environment) was conducted in 
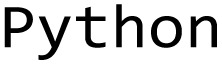
 employing a supervised machine learning approach. Therefore, seven M1 macrophage cells, six of them exhibiting all of the available classes, were classified manually to a certain extent (see Fig. S1A) based on the spatial distribution of the intensity of characteristic Raman modes for lipids^60–64^ and DNA^26,28–30^ (*cf.* demo feature stack images in Fig. S1B). With the aid of this annotated ground truth, a Random Forest classifier was trained on feature maps derived from the integration results of a set of Raman modes that were selected on the basis of spectroscopic considerations. This approach was adopted in order to restrict the number of features while ensuring the inclusion of meaningful content. In light of the aforementioned considerations, it was hypothesized that the following eight integration areas would prove to be promising in terms of achieving optimal outcomes: (789 ± 15) cm^−1^, (1342 ± 15) cm^−1^, (1267 ± 15) cm^−1^, (1100 ± 15) cm^−1^, (1447 ± 15) cm^−1^, (1661 ± 15) cm^−1^, (2850 ± 15) cm^−1^, and (2940 ± 15) cm^−1^. These modes were chosen in a way that tried to omit characteristic Raman bands of the drug BRP-685, which is the C≡N-stretching mode at 2223 cm^−1^ but also the bands at (1510 ± 5) cm^−1^ and (1618 ± 5) cm^−1^ (*cf.* Fig. S7). To further limit the influence of underlying contributions from neighboring bands, the integration result included only the area between the spectrum and a linear function calculated between the margins of the integration interval.

#### Separating cell and background

From the result of the segmentation procedure, a binarized mask was created, which separated the image into the main cellular area and background. Therefore, the segmented image (see segmentation of cell areas) was first thresholded with a lower boundary value of 1.5 (the cellular area was encoded by numeric values: 1 – background, 2 – other cellular matrix area, 3 – nucleus, and 4 – lipid droplets). After an eroding and dilating operation, only the largest region within the mask was kept, which ensured that no partly measured cells at the boundary of the image were included later into the evaluation, if not tightly connected to the main cellular area. Any holes within the final cellular area were eventually removed by a hole-filling algorithm in 
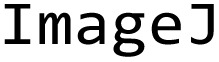
 (version 1.54p). In rare cases, manual adjustments became necessary to exclude high-intensity noise in the background that was misclassified before. In other cases, where the cell boundary was not continuous, and the eroding operation thus killed significant amounts of the cell area, a manual adjustment of the cell boundary followed by a hole-filling step was necessary as an intermediate operation.

#### Comparison of spectral features

The spectral bands were detected in Fig. 2 according to the following procedure. First, the spectra were fitted in 
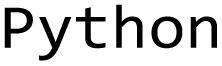
 by a univariate spline (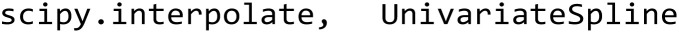) with a spline degree of 4 and a smoothing factor of 1000 to receive a derivable expression of the spectrum. Where a band maximum occurs, the second derivative should show an extreme value, lower than 0. Only those peaks were considered where the extreme passed a height limit, which was 2% of the respective maximum value, to avoid detection of noise. The sensitivity of peak detection was controlled by a *Δ*-parameter (*Δ* = 0.01) that describes the vertical distance that needs to at least be overcome to make a maximum/minimum count. All stated parameters were optimized by visual inspection.

#### Colocalization analysis

The colocalization analysis, as performed in Fig. 3, builds on Schultz & Zopf *et al.* (2024)^39^ and Meyer *et al.* (2013),^38^ but here applied to two bands in the silent wavenumber region. The intensities of the C≡C stretching mode of 17-octadecynoic acid and the C≡N stretching mode of BRP-685 were extracted from the spontaneous Raman spectra by integrating the background-corrected signal within the spectral intervals (2115 ± 15) cm^−1^ (C≡C) and (2223 ± 15) cm^−1^ (C≡N) for each pixel of the hyperspectral data cube. To minimize the interference from residual water contributions, an additional linear background was estimated and subtracted within each integration window. Each integration result was then divided by the mean integration result of the band at (789 ± 15) cm^−1^ (DNA^26,28–30^) in the nucleus region, which served as a reference to correct for variations of the system's performance.^31^

Considering only the pixel values within the identified cell area (see “Separating Cell and Background” for mask generation), the integration-based results were visualized in a scatter plot, where each pixel is represented by its C≡N/reference contribution from BRP-685 on the *x*-axis and its C≡C/reference contribution from 17-ODYA on the *y*-axis (Fig. 4A). In this 2D colocalization plot, pixels with similar signal intensities in both channels cluster together, while those with differing values separate based on the magnitude of variation. Pixels exhibiting consistent C≡C/C≡N ratios align along a straight line through the origin, with the slope corresponding to this ratio (Fig. 4A). The total signal intensity increases along these lines from the lower left to the upper right of the diagonal. Pixels where no colocalization between both analyzed components is observed can be found on or in close proximity to one of the axes, respectively (Fig. 4A). Pixels with negative *x*- (C≡N/reference) and/or the *y*-values (C≡C/reference) were excluded from the scatter plot.

Scatter plots were colored by local point density estimated *via*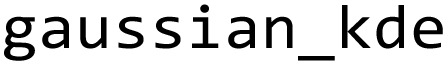 (Fig. 4B). For the creation of Fig. 3B and S9, the scatter plot was divided into four segments, and the pixels belonging to each segment were indicated by the same color when projected back onto the original pixel grid. This back projection allows insights into the spatial assembly of chemically similarly composed pixels. Importantly, each pair of segmentation boxes is separated by a line of common slope (the below or above the central line, respectively). Consequently, the upper pair of segmentation boxes comprised pixels with a higher C≡C/C≡N-ratio (red/orange) as compared to the lower pair (blueish). Within each pair, pixels with lower intensities (dark blue and red) were separated from those with higher intensities (cyan and orange) along the same linear trend (Fig. 4B). The position of the lower-intensity cluster was determined by the density coloring of the entire scatter plot, with the upper boundary of the lower-intensity segmentation box set just beyond the point of highest density (*cf.*Fig. 3 and 4B).

#### Evaluation of the SRS data

Spectral datasets were processed by extracting pump and Stokes power values from the metadata file to calculate correction factors compensating for power fluctuations. The spectral intensity data were then corrected and normalized to a *y*-axis range of 0–1 within the 2050–2300 cm^−1^*x*-axis range.

For the SRS images presented in Fig. 3, all displayed images were obtained by subtracting an off-resonance image recorded at 2500 cm^−1^ to remove background contributions.

## Author contributions

Conceptualization: C. S., P. M. J., P. D., provision of study materials: Z. T. G. M., E. B., investigation: C. S., P. D., data analysis and interpretation: C. S., P. M. J., P. D., visualization: C. S., P. M. J., methodology: C. S., P. M. J., P. D., supervision: P. M. J., T. M.-Z., M. S., O. W., J. P., writing – original draft: C. S., P. M. J., writing – review & editing: all authors, funding acquisition: O. W., J. P.

## Conflicts of interest

There are no conflicts to declare.

## Supplementary Material

SC-OLF-D5SC09493C-s001

## Data Availability

Raw research data of the spontaneous Raman and SRS measurements for this paper as well as the used classifier for the cell segmentation are available at https://doi.org/10.5281/zenodo.17151831. Supplementary information (SI) is available. See DOI: https://doi.org/10.1039/d5sc09493c.
