# Supplementary material for: Unveiling the molecular dynamics of a nitrile-containing 5-lipoxygenase-activating protein antagonist in primary macrophages through Raman spectroscopy
Source: Chem Sci. 2026 Jan 9;17(3):1613–23. doi: 10.1039/d5sc09493c (PMC12784294; doi:10.1039/d5sc09493c)
Supplement: SC-017-D5SC09493C-s001 [file SC-017-D5SC09493C-s001.pdf]

Supplementary Information (SI) for Chemical Science.
This journal is © The Royal Society of Chemistry 2025

Supporting Information

Unveiling the Molecular Dynamics of a Nitrile-containing 5-Lipoxygenase-activating Protein Antagonist in Primary Macrophages through Raman Spectroscopy

Constanze Schultz [a], Paul Mike Jordan [b], Philipp Dahlke [b], Zehra Tuğçe Gür Maz [c], Erden Banoğlu [c], Tobias Meyer-Zedler [a], Michael Schmitt [d], Oliver Werz [b] and Juergen Popp [a,d,*]

[a] Leibniz Institute of Photonic Technology (Leibniz-IPHT), Member of Leibniz Health Technologies, Member of the Leibniz Center for Photonics in Infection Research (LPI), Albert-Einstein-Str. 9, 07745 Jena, Germany

[b.] Institute of Pharmaceutical Chemistry, Friedrich Schiller University Jena, Philosophenweg 14, 07743 Jena, Germany

[c.] Department of Pharmaceutical Chemistry, Faculty of Pharmacy, Gazi University, Taç Sok. No:3 Yenimahalle 06560 Ankara, Turkey

[d.] Institute of Physical Chemistry (IPC) and Abbe Center of Photonics (ACP), Member of the Leibniz Center for Photonics in Infection Research (LPI), Friedrich Schiller University Jena, Helmholtzweg 4, 07743 Jena, Germany

[*] Correspondence may be addressed to Juergen Popp (juergen.popp@uni-jena.de)

Table of Contents

Supporting Tables....2
- Detailed parameters for the spontaneous Raman measurements....2

Supporting Figures....3
- BRP-685 synthesis route....3
- Cell segmentation....4
- Validation of the segmentation....5
- Mean BRP-685 uptake based on strength of the CN-stretching band....5
- Distribution of BRP-685 based on the presence of the CN-stretching band....6
- Analysis of the depth profile....12
- DFT calculations on BRP-685....13
- Colocalization analysis....14

Supporting Tables

Detailed parameters for the spontaneous Raman measurements

Table S 1 Overview of the samples analyzed with spontaneous Raman microspectroscopy

treatment	substrate	positions measured	Integration
M1 30 µM BRP-685	CaF_2	5	0.5 s px^{-1}
		1	1.0 s px^{-1}
M2 30 µM BRP-685	CaF_2	5	0.5 s px^{-1}
		2	1.0 s px^{-1}
M1 0 µM BRP-685	CaF_2	5	0.5 s px^{-1}
		1	1.0 s px^{-1}
M2 0 µM BRP-685	CaF_2	5	0.5 s px^{-1}
		1	1.0 s px^{-1}
M1 3 µM BRP-685	CaF_2	5	1.0 s px^{-1}
M1 3 µM BRP-685	CaF_2	5	1.0 s px^{-1}
M1 3 µM BRP-685	CaF_2	5	1.0 s px^{-1}
M2 3 µM BRP-685	CaF_2	6	1.0 s px^{-1}
M2 3 µM BRP-685	CaF_2	5	1.0 s px^{-1}
M2 3 µM BRP-685	CaF_2	5	1.0 s px^{-1}
M1 30 µM BRP-685<br>417 µM 17-ODYA	glass	5	1.0 s px^{-1}

Supporting Figures

BRP-685 synthesis route

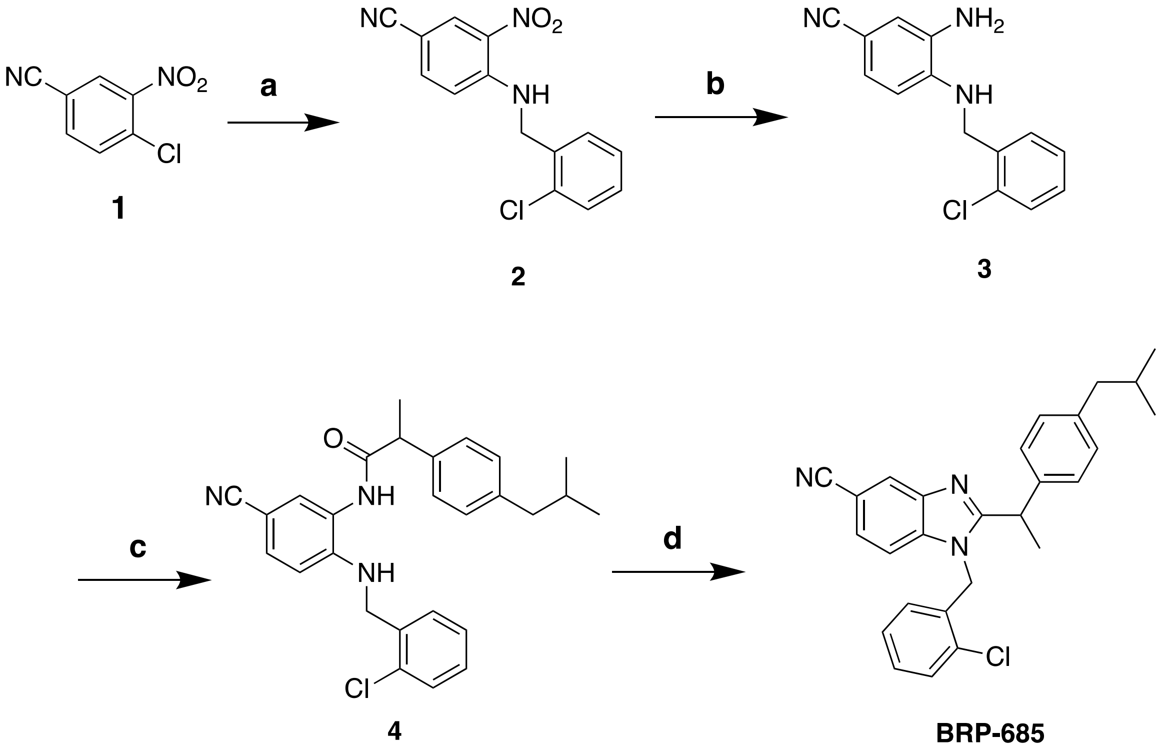

Scheme S1. Synthesis of BRP-685. Reagents and conditions: (a) 2-chlorobenzylamine, MeOH; (b) SnCl2, EtOH; (c) ibuprofen acyl chloride, DIEA, DCM; (d) Dioxan, HCl, reflux.

Cell segmentation

A

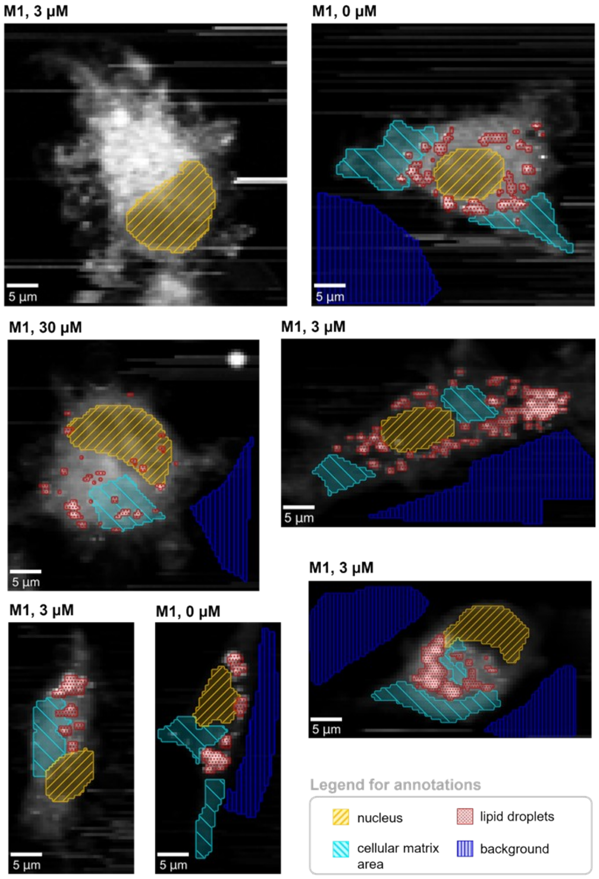

B

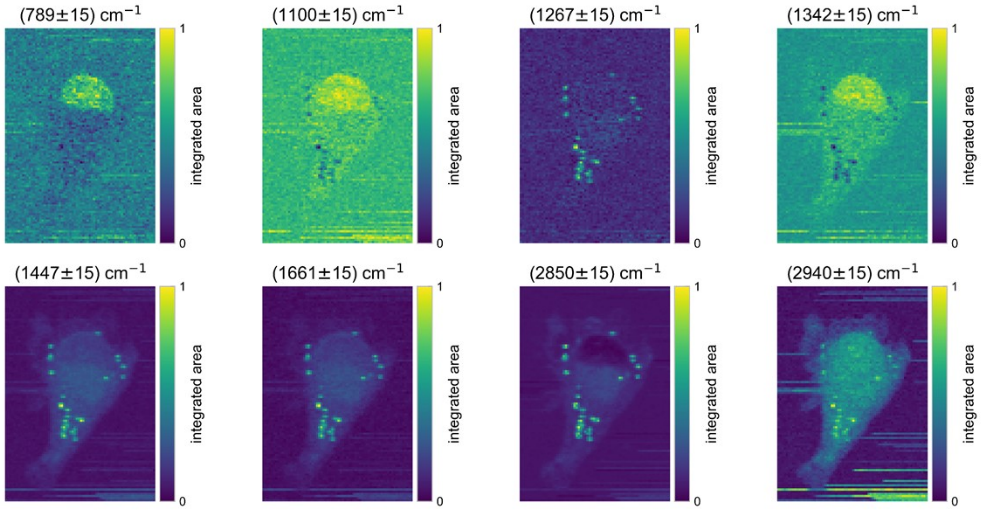

Figure S 1 A Annotated images for training the Random Forest Classifier. The ground truth is indicated by colored areas and projected on the integration result of the spectral region (2850 ± 30) cm^{-1}. All cells subjected to the classification were measured via spontaneous Raman microspectroscopy. **B** ntegrations across several bands of the spontaneous Raman spectrum. The selected spectral regions were used to generate the feature stacks. The resulting images clearly reveal lipid-rich regions as well as the nucleus. Shown as an example is an untreated control cell.

Validation of the segmentation

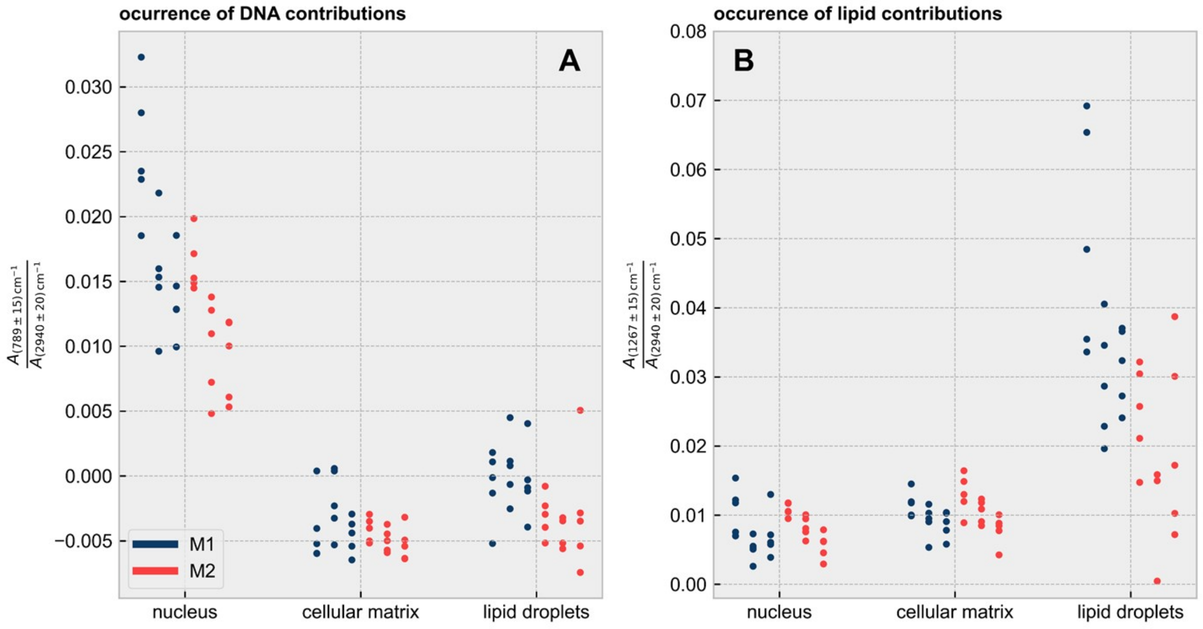

Figure S 2 Verification of the segmentation procedure of BRP-685-treated cells (3 µM) imaged via spontaneous Raman scattering. For the segmented areas within the entire cell region the intensity of different Raman bands was evaluated. To allow for a comparison between different individual cells and batches, the respective signals were divided by the protein signal which was approximated by an integration between the area under the curve and the abscissa of the plot in the interval (2940 ± 20) cm^{-1}. Each column of points within a class represents a batch of cells, while each point represents a measured cell. **A** evaluates the content of the band at (789 ± 15) cm^{-1} which is a marker for the presence of DNA. High contents are in particular present in the nucleus as expected. **B** analyses the presence of saturated lipids ((1267 ± 15) cm^{-1}). High abundance of the respective Raman band appears in particular within the lipid droplets.

Mean BRP-685 uptake based on strength of the CN-stretching band

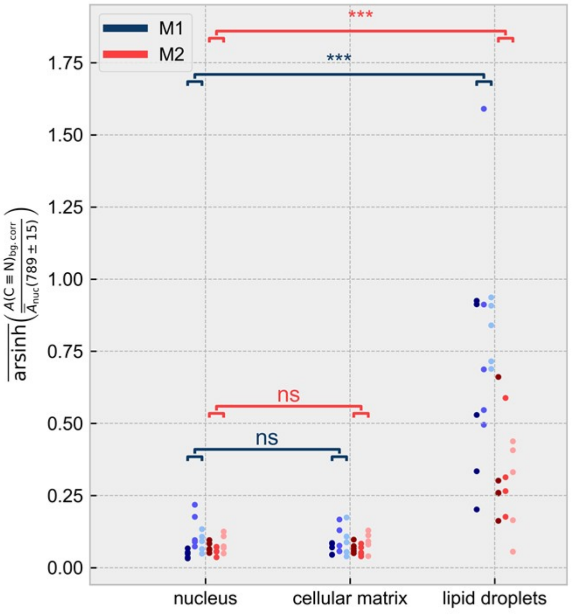

Figure S 3 Visualization of the mean value of the segmented regions per cell in the spontaneous Raman microspectroscopic measurements. Cells of the same batch are shown in the same color. For the determination of the significance, a Wilcoxon signed-rank test was performed (ns: not significant, $p > 0.05$; ***: highly significant, $p < 0.001$). Shown are results for cells treated with BRP-685 (3 µM) and imaged via spontaneous Raman scattering. For details on the choice of the displayed y-axis band ratio, please refer to the main text.

Distribution of BRP-685 based on the presence of the CN-stretching band

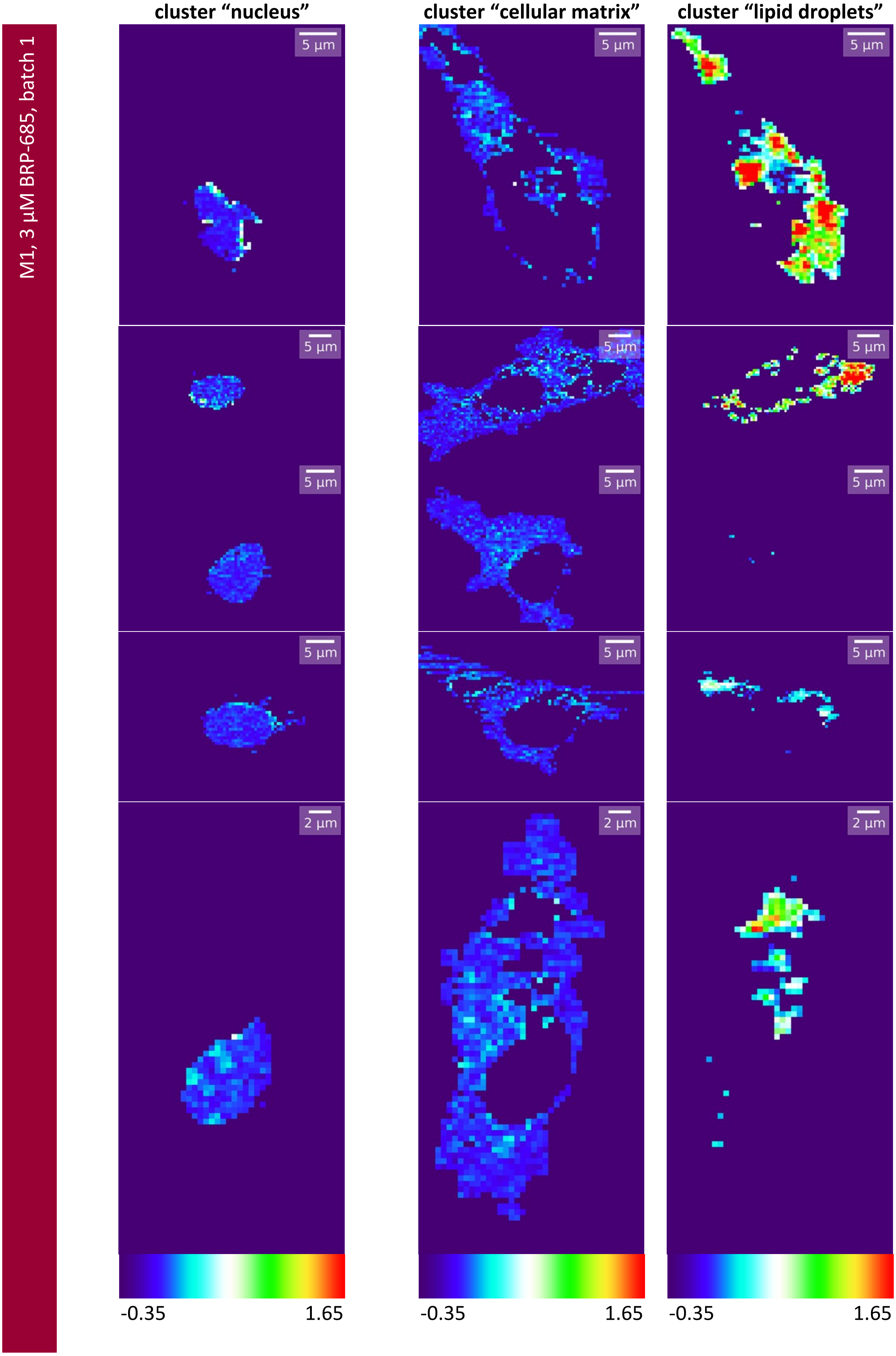

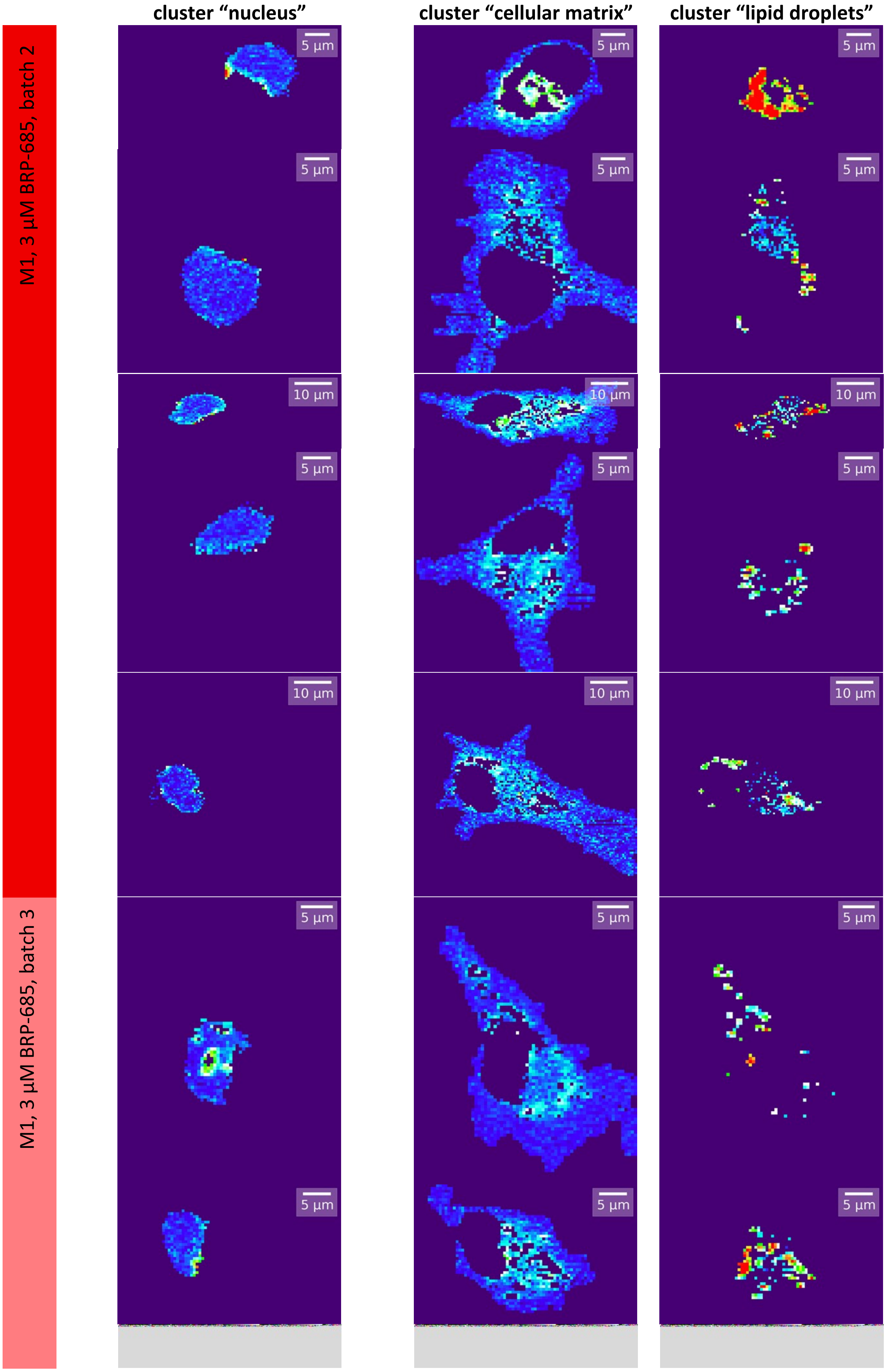
cluster "nucleus"
cluster "cellular matrix"
cluster "lipid droplets"
M1, 3 µM BRP-685, batch 2
M1, 3 µM BRP-685, batch 3
5 µm
10 µm

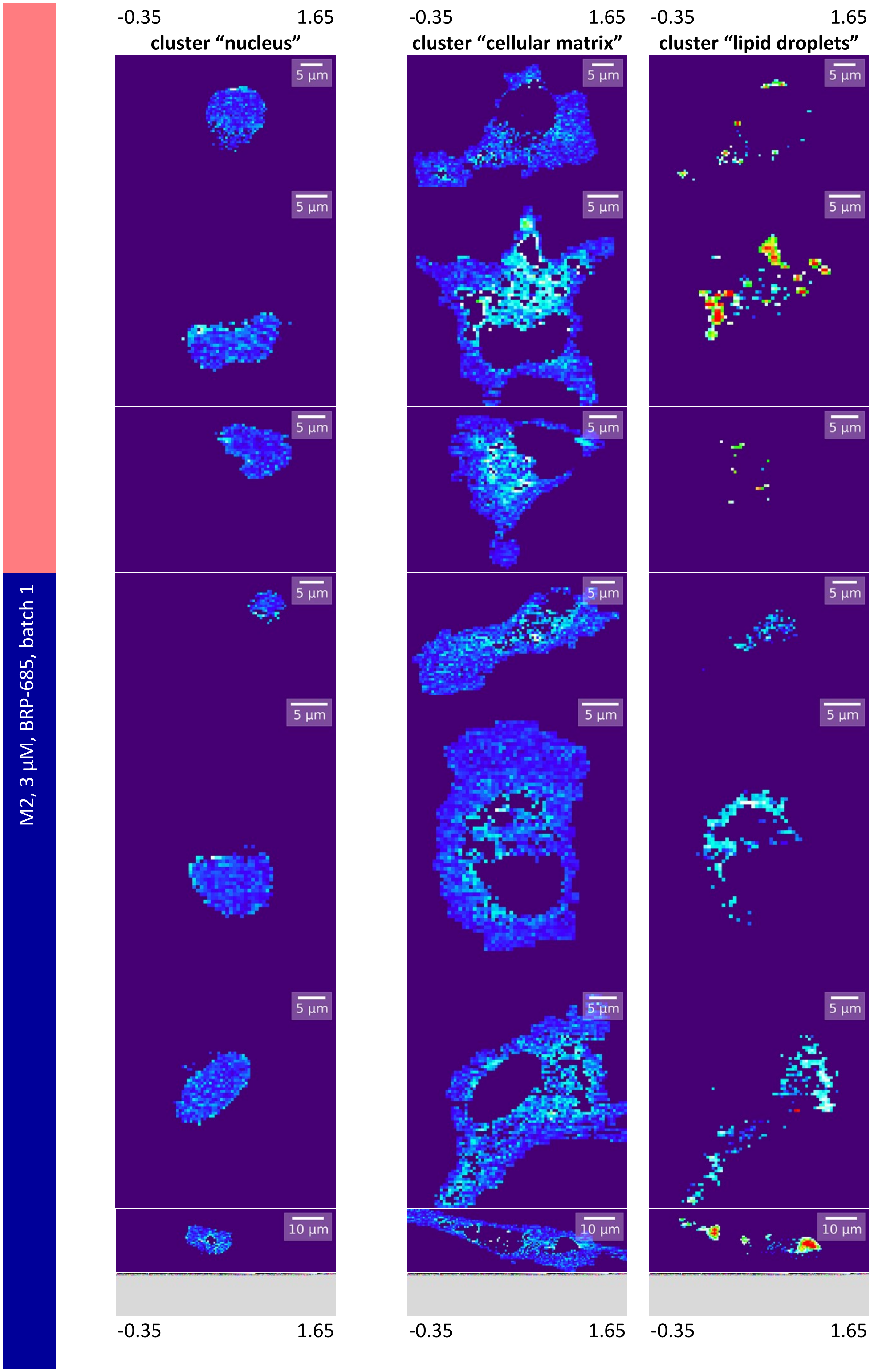

-0.35
1.65
cluster “nucleus”
cluster “cellular matrix”
cluster “lipid droplets”
5 µm
10 µm
M2, 3 µM, BRP-685, batch 1

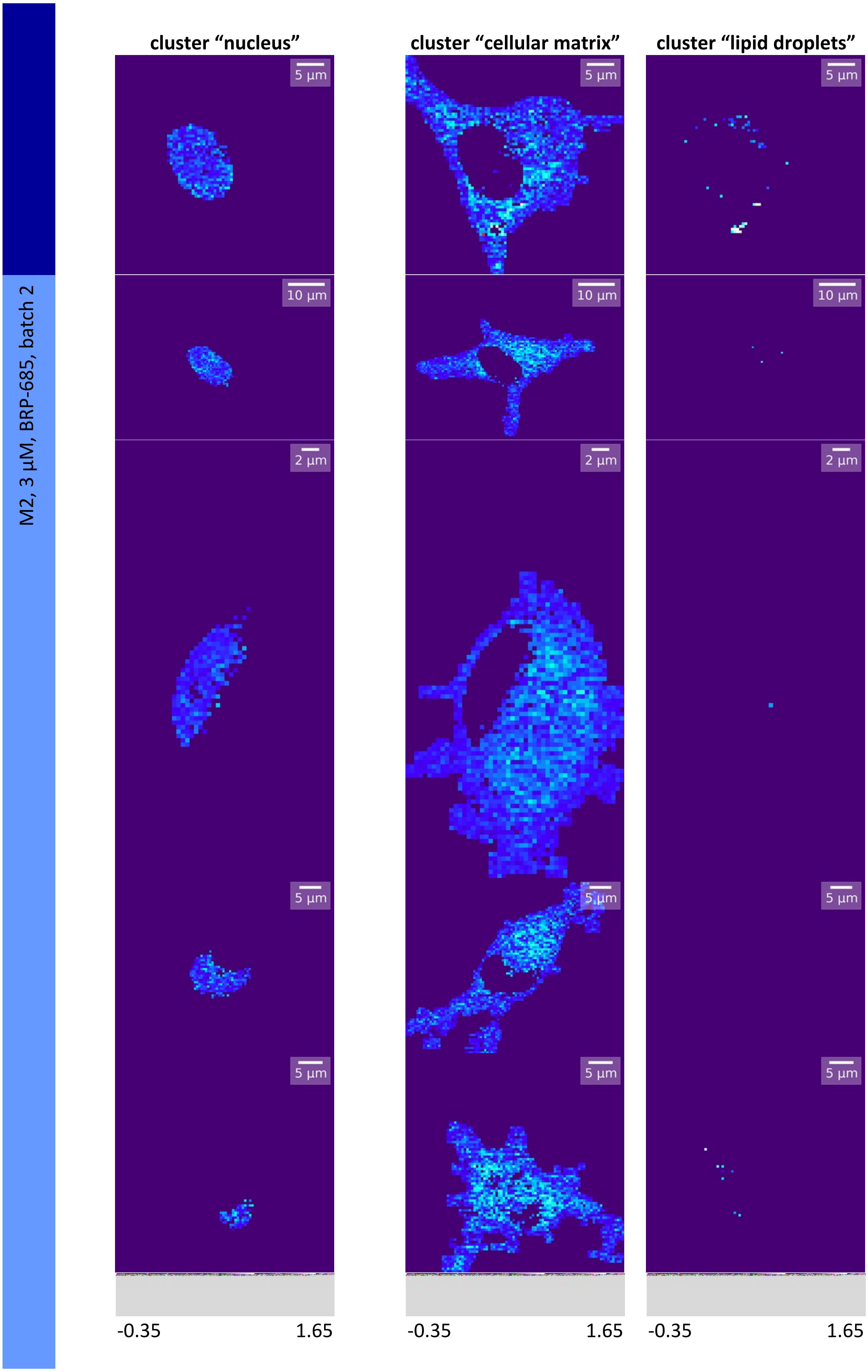
M2, 3 µM, BRP-685, batch 2
cluster "nucleus"
cluster "cellular matrix"
cluster "lipid droplets"
5 µm
10 µm
2 µm
-0.35
1.65

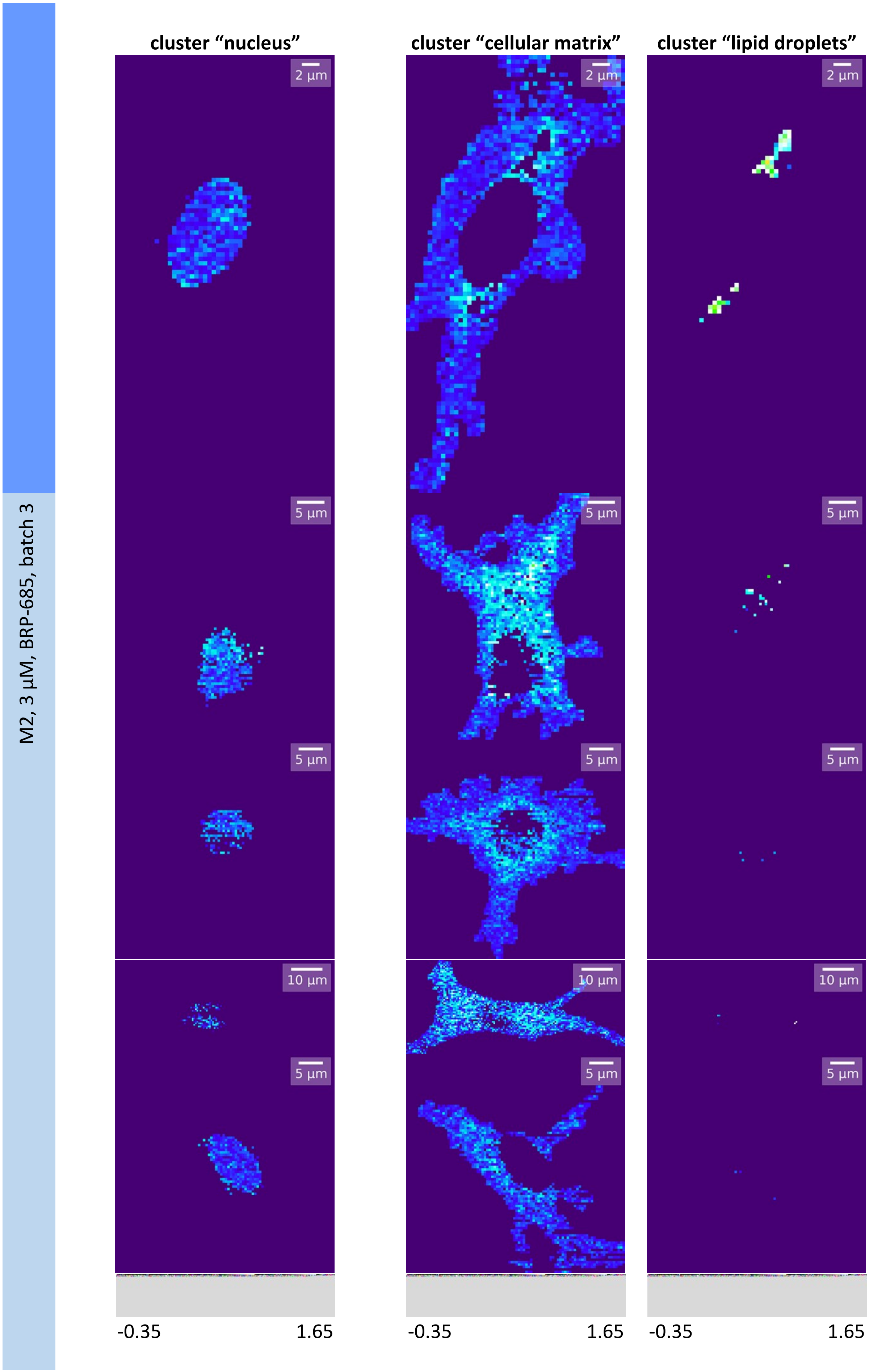

M2, 3 µM, BRP-685, batch 3
cluster "nucleus"
cluster "cellular matrix"
cluster "lipid droplets"
2 µm
5 µm
10 µm
-0.35
1.65

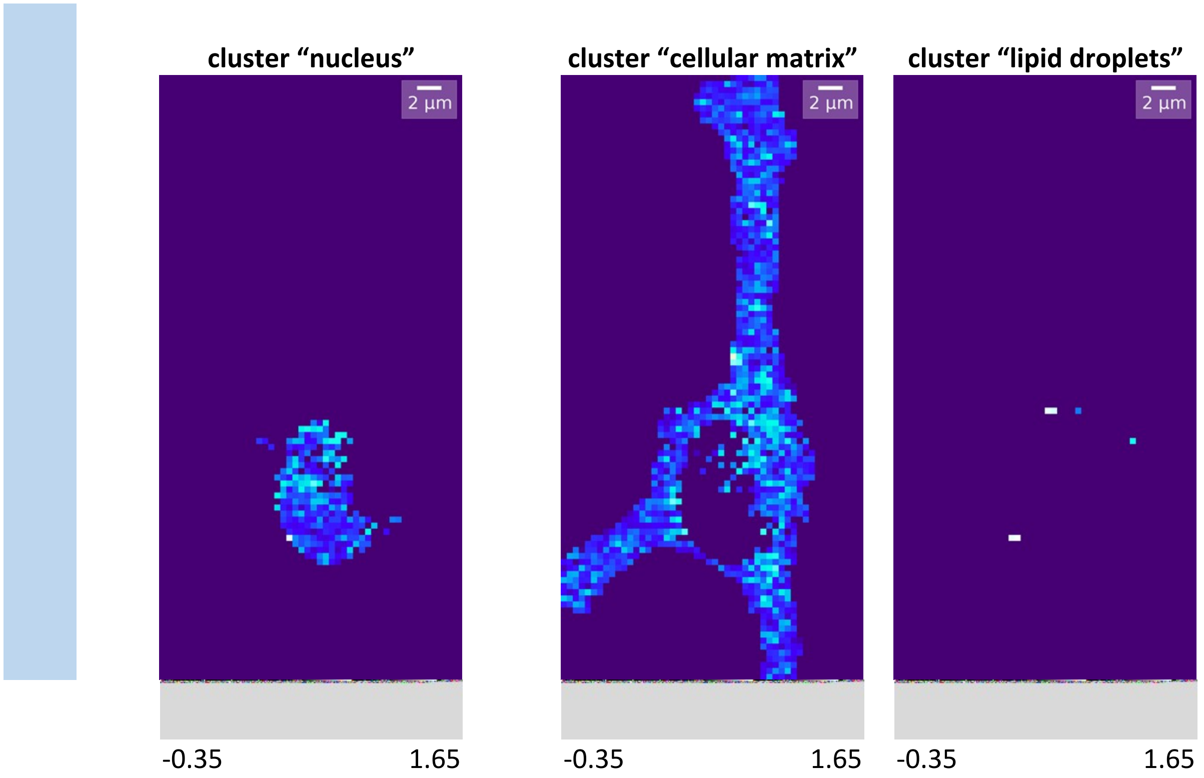

Figure S 4 Visualization of the ratio $arsinh\left(\frac{A(C\equiv C)_{bg}}{\overline{A}_{nuc}(789\pm 15)}\right)$ in the segmented areas of BRP-685-treated cells (3 µM), recorded via spontaneous Raman microspectroscopy and used for the creation of Figure 1C. The color map of all images is scaled between -0.35 and 1.65. C Cells from the same batch are represented by a uniform color in the first column, with different batches distinguished by distinct colors: M1 macrophages are shown in shades of red, M2 macrophages in shades of blue. Subsequent columns show segmented regions corresponding to “nucleus,” “cellular matrix,” or “lipid droplet” clusters. This panel, complementing Figure 1C, illustrates both the spatial distribution of pixels and regions with elevated BRP-685 content and the accuracy of the cell segmentation. For details on the choice of the displayed band ratio, please refer to the main text.

Analysis of the depth profile

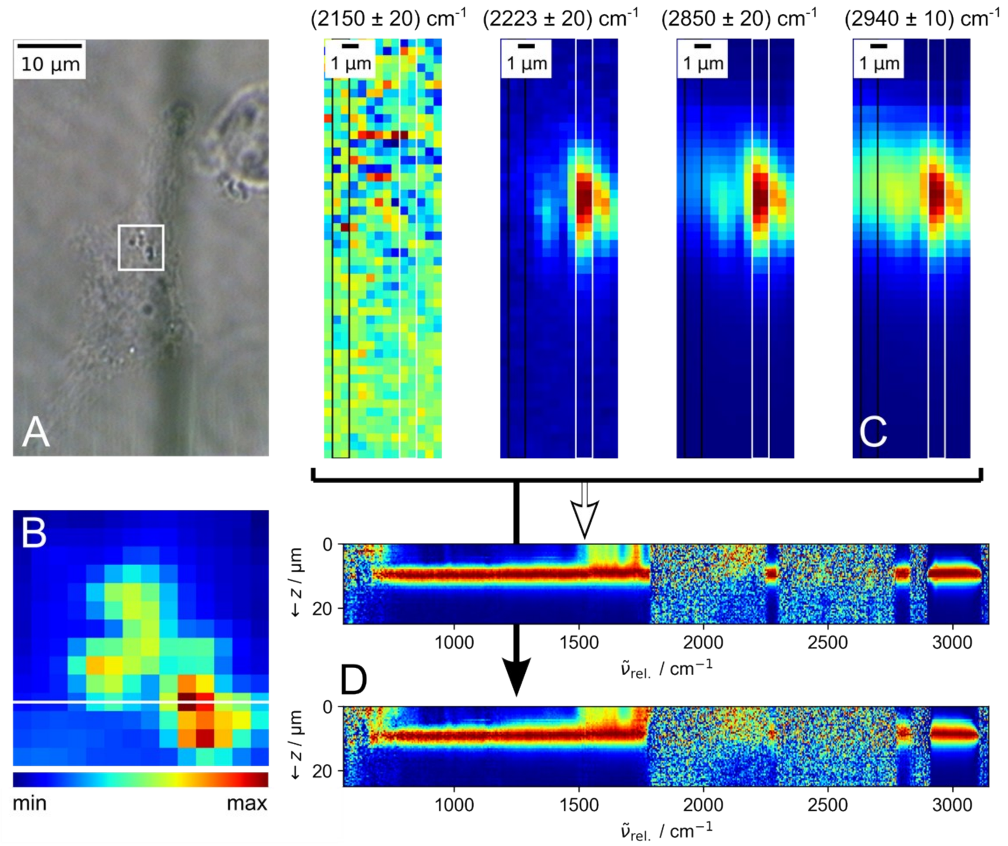

Figure S 5: Investigating the depth profile of selected spontaneous Raman signatures of BRP-685-treated M1-cells (3µM). **A** Bright-field image of an M1 cell with the analyzed region indicated by a white square. **B** Integration image across the wavenumber range (2850 ± 20) cm^{-1} (corresponding to the CH_2-stretching mode) for an arbitrary slice of the measured stack. The signal relates to the presence of lipid droplets **C** Depth profiles reconstructed along the white line in B, showing integration images for four spectral regions as indicated in the figure. The colors were scaled between the minimum and maximum value per integration image. For integrations in the silent wavenumber region, an additional background correction was applied on top of the SNIP correction (see Methods). The additional background estimation involved fitting a linear baseline between the integration limits to suppress signal contributions from the water background. **D** Wavenumber–depth heatmaps generated at the positions marked in C (2×2 pixel columns). The CN-mode signal aligns with characteristic cell signals, indicating that the inhibitor is taken up by the cell rather than remaining on its surface. To ensure a consistent color scale, the depth-dependent signal at each wavenumber position was normalized individually between its local minimum and maximum values.

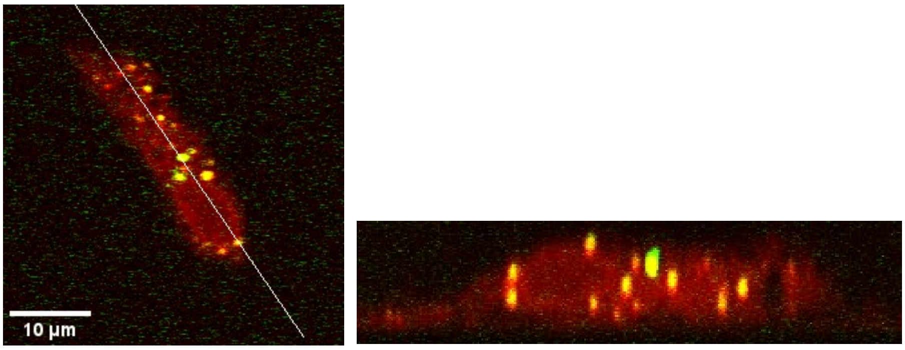

Figure S 6: SRS overlay image of BRP-685-treated cells (30 µM), recorded at 2930 cm^{-1}(red) and 2223 cm^{-1} (green). As the cross-section view (along the white line) shows, the BRP-685 accumulation spots are within the cell area, which verifies that BRP-685 is taken up instead of particles stuck to the cell surface.

DFT calculations on BRP-685

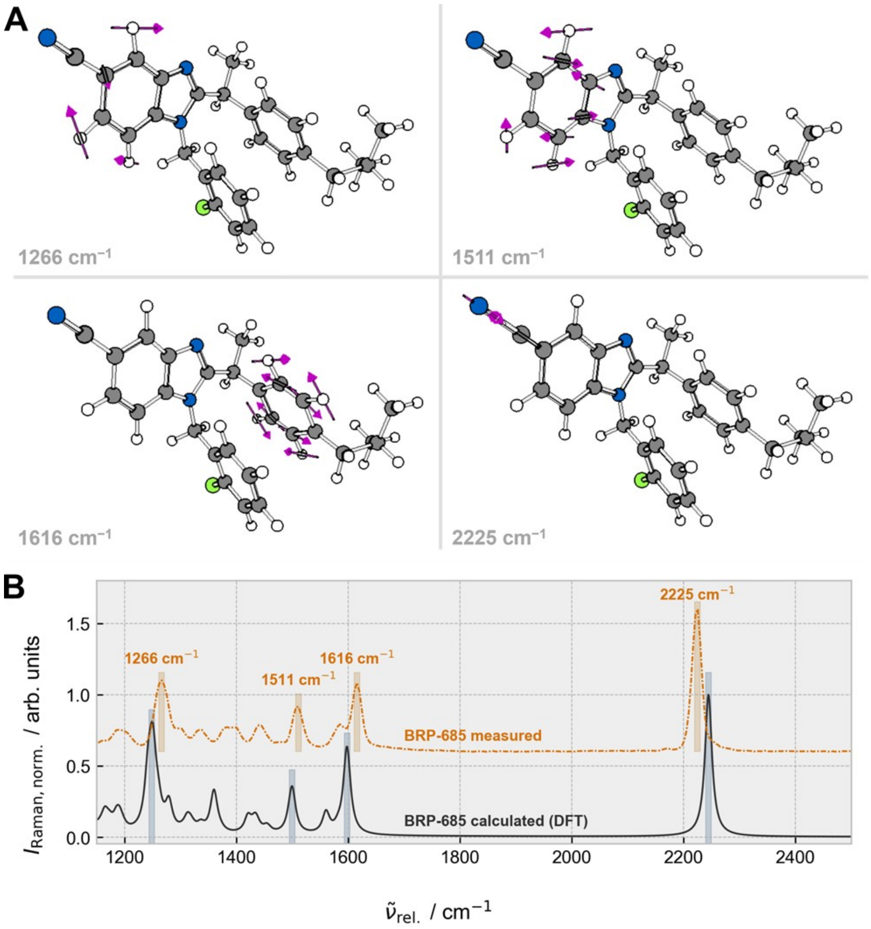

Figure S 7 Molecular movement for the most intense Raman bands at 1266 cm^{-1}, 1511 cm^{-1}, 1616 $cm^{-1,}$ and 2225 cm^{-1} of BRP-685. **A** The scaled vibrational displacement vectors were displayed within ChemCraft[1] and shown in purple with a visualization threshold of 0.2 within this figure. While the intense vibrational mode in the wavenumber silent region can be attributed to the stretching vibration of C≡N, all other intense modes arise from vibrations of the aromatic cores of the molecule. Atoms were colored by element (gray: carbon, blue: nitrogen, white: hydrogen, green: chlorine). **B** displays the measured (orange) and DFT-calculated (black) Raman spectrum of BRP-685. The DFT calculation was necessary to get insights into the molecular movements of the most intense observed Raman bands. A scaling factor of 0.9626 was used for the correction of the DFT-calculated frequency axis. More details on the DFT-calculation are provided in the Experimental Section. The spontaneous Raman spectrum shown in B was obtained from a particle in the cell culture.

SRS large area scan

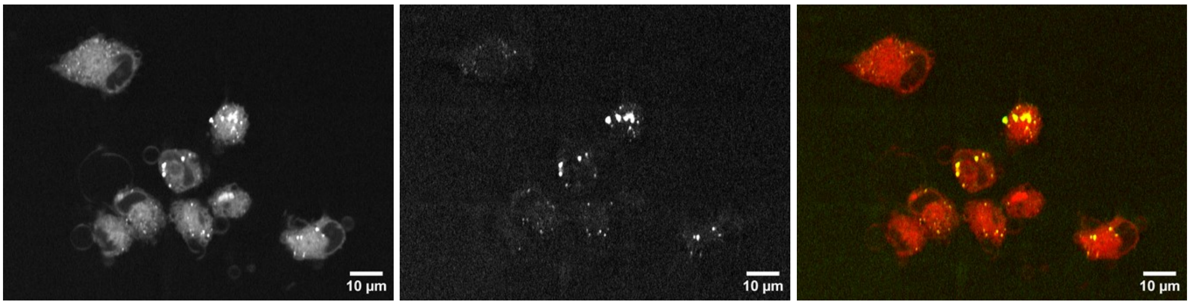

Figure S 8: SRS images of BRP-685 treated cells (30 µM), recorded at 2850 cm^{-1}(left) and 2223 cm^{1}(middle), and the respective overlay (red: 2850 cm^{1}, green: 2223 cm^{1}). While the left image shows the presence of CH_2-bonds, the middle image visualizes the distribution of BRP-685, which can be found to the largest extent in the lipid droplet regions.

Colocalization analysis

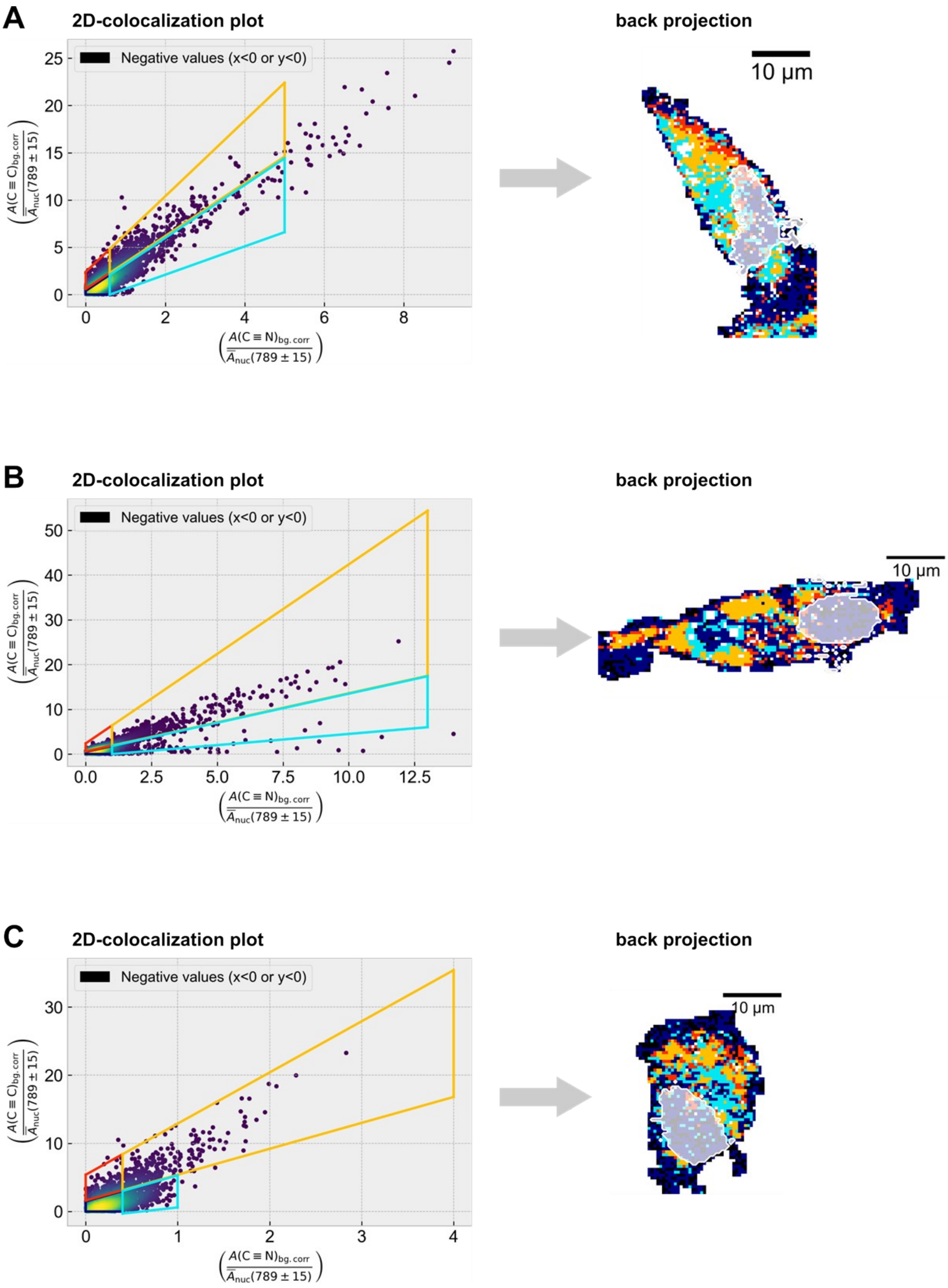

Figure S 9: Colocalization analysis of the C≡C (17-ODYA) and C≡N (BRP-685) bands based on spontaneous Raman spectra, as described in the Methods section. In the back projection image, pixels were colored according to the corresponding segments in the scatter plot. Pixels with negative values in either the x- or y-coordinate of the scatter plot are shown in black. The nucleus is indicated by a semi-transparent white contour overlaid on the back-projection image.

References

[1] G. A. Zhurko, "Chemcraft. graphical program for visualization of quantum chemistry computations". Version 1.8, build 682, **2005,** can be found under https://www.chemcraftprog.com (accessed: 11/25/2024).
